# A tale of textiles: Genetic characterization of historical paper mulberry barkcloth from Oceania

**DOI:** 10.1371/journal.pone.0233113

**Published:** 2020-05-18

**Authors:** Bárbara Peña-Ahumada, Mónica Saldarriaga-Córdoba, Olga Kardailsky, Ximena Moncada, Mauricio Moraga, Elizabeth Matisoo-Smith, Daniela Seelenfreund, Andrea Seelenfreund

**Affiliations:** 1 Departamento de Bioquímica y Biología Molecular, Facultad de Ciencias Químicas y Farmacéuticas, Universidad de Chile, Santiago, Chile; 2 Centro de Investigación en Recursos Naturales y Sustentabilidad, Universidad Bernardo O’Higgins, Santiago, Chile; 3 Department of Anatomy, University of Otago, Dunedin, New Zealand; 4 Centro de Estudios Avanzados en Zonas Áridas (CEAZA), La Serena, Chile; 5 Programa de Genética Humana, Instituto de Ciencias Biomédicas, Facultad de Medicina, Universidad de Chile, Santiago, Chile; 6 Departamento de Antropología, Facultad de Ciencias Sociales, Universidad de Chile, Santiago, Chile; 7 Escuela de Antropología, Facultad de Ciencias Sociales, Universidad Academia de Humanismo Cristiano, Santiago, Chile; Federal University of Mato Grosso do Sul, BRAZIL

## Abstract

Humans introduced paper mulberry (*Broussonetia papyrifera*) from Taiwan into the Pacific over 5000 years ago as a fiber source to make barkcloth textiles that were, and still are, important cultural artifacts throughout the Pacific. We have used *B*. *papyrifera*, a species closely associated to humans, as a proxy to understand the human settlement of the Pacific Islands. We report the first genetic analysis of paper mulberry textiles from historical and archaeological contexts (200 to 50 years before present) and compare our results with genetic data obtained from contemporary and herbarium paper mulberry samples. Following stringent ancient DNA protocols, we extracted DNA from 13 barkcloth textiles. We confirmed that the fiber source is paper mulberry in nine of the 13 textiles studied using the nuclear ITS-1 marker and by statistical estimates. We detected high genetic diversity in historical Pacific paper mulberry barkcloth with a set of ten microsatellites, showing new alleles and specific genetic patterns. These genetic signatures allow tracing connections to plants from the Asian homeland, Near and Remote Oceania, establishing links not observed previously (using the same genetic tools) in extant plants or herbaria samples. These results show that historic barkcloth textiles are cultural materials amenable to genetic analysis to reveal human history and that these artifacts may harbor evidence of greater genetic diversity in Pacific *B*. *papyrifera* in the past.

## Introduction

The reconstruction of the dispersal history of human population in the Pacific has been advanced using the commensal approach. This refers to the study of species living in close association to humans, and that were dependent on humans for their dispersal into Oceania [1]. Based on this direct association, researchers recognized that tracking the movement of these transported species provided a proxy for the movement of prehistoric human populations in the region. Further, by comparing the commensal data with other forms of archaeological, genetic, and linguistic evidence the complexities of human history in the Pacific could be encompassed [1].

Several authors have addressed the non-intentional and intentional dispersal of diverse organisms closely associated to humans. The study of unaware dispersal of microorganisms (*Helicobacter pylori*) [2], the presumably non-intentional dispersion of geckos and skinks [3,4], and the deliberate dispersion of snails [5], Pacific rats, dogs, chicken, breadfruit, kava, and bananas, among others [6] have contributed to construct a coherent, yet multifaceted scenario. For example, the discovery of direct parallels between the linguistic structure of the Pacific languages and the dispersion of different *H*. *pylori* strains is remarkable [7]. Taken together, the study of a number of commensal species has provided insight into the complexity of this entire migratory process, since each species has proven to have a different dispersal history.

Prior to the arrival of Europeans, Pacific peoples manufactured cloth and other textiles using the bark from several plant species such as *Artocarpus altilis*, *Ficus sp*. and *Pipturus sp* [8]. The source of the most valuable textiles in Remote Oceania was the inner bark obtained from paper mulberry (*Broussonetia papyrifera* (L.) L’Hér. ex Vent.), one of the “canoe plants” introduced by the early Austronesian-speaking prehistoric voyagers from Taiwan [9]. On some Pacific islands, barkcloth textiles still maintain great cultural value, because they are part of important ceremonies and rituals associated with all stages of life through death [8, 10]. In Polynesia, barkcloth textiles called tapa or kapa (in Hawaii) have largely been replaced by western cloth, although in the last few years tapa has seen a significant revival on some islands. In traditional contemporary Polynesian cultures, such as in Tonga and Fiji, tapa is still used to wrap newborns, to preserve the vital energy that connects the child to the other world. For weddings, large pieces of tapa are displayed and lain on the pathway, over which the bride and groom will walk after being married. Finally, for funerals, tapa pieces are used to wrap the body of the deceased before burial. Tapa textiles reflect power and prestige, are precious objects to be exhibited, used, handed over as ceremonial gifts and passed on from generation to generation [8, 10]. Pacific dignitaries gifted great numbers of tapa textiles to European explorers of the eighteenth and nineteenth-century expeditions, and some of these are housed today in museums around the world [8].

With the advent of modern molecular genetics, and particularly with the development of Polymerase Chain Reaction (PCR) techniques and ancient DNA (aDNA) protocols, many kinds of cultural materials of animal and plant origin have emerged during the last 20 years as novel sources of genetic information. For example, DNA has been extracted from cultural materials such as papyri [11], wood from a sunken ship [12], feather cloaks [13], parchment [14] and leather [15]. We previously reported DNA extraction from contemporary barkcloth samples, proving that it is possible to amplify DNA from this material [16], despite harsh manufacturing procedures, which involve prolonged beating of the inner bark fibers, sometimes soaking in seawater and final felting or joining of layers and pieces to form large textiles. In the felting process, several layers of bark from different individual plants are integrated into one textile [see 8, 17, 18, 19 for more details of this process]. The recovery and genetic analysis of tandemly repeated ribosomal DNA (rDNA) sequences of a 200 years old archaeological tapa bundle found in a cave on Agakauitai Island, in the Gambier Archipelago, showed that this barkcloth was made of *B*. *papyrifera* fiber [20].

The process of Austronesian expansion in the Pacific comprised human-mediated dispersal of many plant species, including paper mulberry. We have used this fiber plant as a proxy to reconstruct the dispersion of plants and humans in Oceania [9, 21, 22, 23, 24, 25]. Our previous studies of *B*. *papyrifera* plants comprised those Pacific islands where extant plants are still present or from available herbarium samples.

In this paper, we characterize the genetic makeup of 13 barkcloth textiles from different locations (Rapa Nui, Hawaii, New Guinea, Fiji, American Samoa and Gambier Is.) in order to answer three central questions of curators, Pacific researchers and the Pacific communities at large: 1) Which plant species were used as source of fiber? 2) Can we identify alleles that had not been observed previously in herbaria or contemporary samples? 3) Can we detect genetic footprints that allow assignment of un-provenanced textiles to specific islands?

Due to their great cultural significance and the traditional practice of keeping and handing down tapa textiles for generations, and their presence in museum collections around the world, they represent a novel and relevant source of DNA to investigate the genetic diversity and dispersal of this plant in the Pacific. Some of the existing pieces may predate even the earliest paper mulberry herbaria samples from the region. In addition, barkcloth textiles may be available from islands where the plant has disappeared and where herbarium records are absent, as in the case of the Gambier Islands.

## Materials and methods

### Barkcloth samples

**Fig 1:**
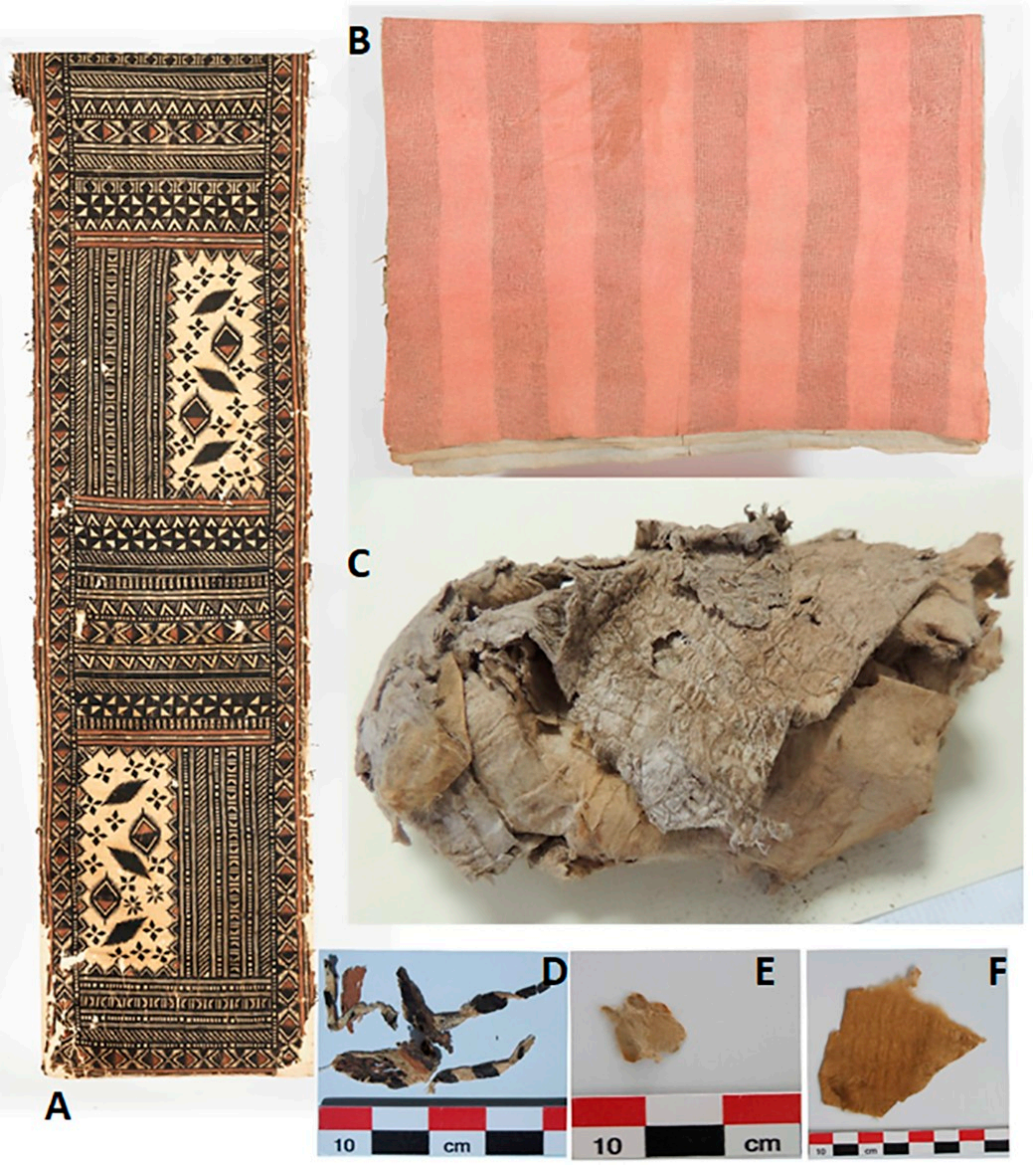
Examples of barkcloth textiles sampled for this study. (A) Painted tapa from Fiji (1928, Honolulu Museum of Art Catalogue #2506). Donated to the Museum in 1928. Size of tapa 61.6 x 350.5 cm. (B) Multilayered bed tapa (Kapa Moe) from Hawaii (Honolulu Museum of Art, Catalogue #662.1). Size: 208 cm x 281 cm. (C) Gambier Islands textile from a funerary context. Four bags containing large number of pieces that range in size from a few cm in length and width to approximately 15 x 25 cm. Image of contents of Bag A. (D) Sample BQUCHTE008 taken from the Fijian tapa (Fig 1A). E. Sample BQUCHTE005 was taken from a Hawaiian tapa piece (Fig 1B). (F) Sample BQUCHTE016 taken from the Gambier Island tapa [20], Bag B2. Scale bar divisions: 1 cm.

We initiated the present study with a set of 16 samples taken from thirteen very dissimilar barkcloth textiles from five Pacific islands or island groups, all manufactured between the nineteenth and the end of the twentieth century. Some of the pieces have no known date of manufacture or a doubtful provenance. One of these textiles is from an archaeological context in the Gambier archipelago and the four samples from this barkcloth have been analyzed only with the nuclear Internal Transcribed Sequence marker (ITS-1) [see 20] (Table 1 and Figs 1 and S1 and S1 Text). Export of samples from Tahiti to Chile was possible through permit 548/2014 issued by the Service de la Culture et du Patrimoine of French Polynesia to AS. Other samples were provided by the respective institutional curators or private owners. All necessary permits were obtained for the described study, which complied with all relevant regulations. Specimen accession numbers are given in Table 1 when available.

**Table 1:** Barkcloth textile samples included in this study. Sample code, locality, date of manufacture, and available data from the collection.

Sample code	Locality	Manufacturing date	Collection data, provenance description and additional information
BQUCHTE001	Rapa Nui (?)	Not known	B.P. Bishop Museum, Hawaii. Catalogue number D. 2228* attributed to Easter Island. Brown painted tapa with three rows of stitched border with white cordage. Sample is from the cordage.
BQUCHTE002	Hawaii	Not known	B.P. Bishop Museum, Hawaii. Catalogue #2494 small Hawaiian tapa sampler
BQUCHTE003	Hawaii (?)	Not known	B.P. Bishop Museum, Hawaii. Small tapa sample. “No water mark” written on envelop. No catalogue number, probably Hawaii.
BQUCHTE004	New Guinea	1980–1990	Painted Maisin tapa, Oro Province, New Guinea, dated to aprox. 1980–1990 Private collection T. Allen
BQUCHTE005	Hawaii	Not known	Honolulu Museum of Art, Hawaii. Catalogue number 622.1 Bed tapa (kapa moe). Five layers of tapa, outer layer plane pink alternating with bands of small black square dots closely stamped together. Inner layers of tapa are not dyed. Sample is from the inner layers. (see Fig 1B)
BQUCHTE006	Hawaii	Not known	Honolulu Museum of Art, Hawaii, Catalogue #4121. Outer layer of a brown bed tapa (kapa moe) or a tapa hanging. Decorated all over with a geometric design. Pattern in darker brown. Lined with brown muslin and backed with a watermarked yellow sheet. Sample taken from the outer layer.
BQUCHTE007	Fiji	Around 1970	Fiji 1970 T3 Private collection T. Allen
BQUCHTE008	Fiji	Before 1928	Honolulu Museum of Art. Hawaii. Accession #2506 Donated to the Museum in 1928. Size 61.6 x 350.5 cm (see Fig 1A)
BQUCHTE009	Hawaii (?)	Not known	B.P: Bishop Museum, Hawaii catalogue number 2492. Small Hawaiian tapa sample
BQUCHTE010	Hawaii (?)	Not known	B.P. Bishop Museum. No accession number. Small Hawaiian tapa sample. No additional information.
BQUCHTE011	Hawaii (?)	Not known	B.P. Bishop Museum, Hawaii. Emory collection? Catalogue #T8 Small tapa sample, probably from Hawaii.
BQUCHTE012	American Samoa	Around 1880–1900	Private collection T. Allen. Tapa from American Samoa, dated to 1880–1900
BQUCHTE013	Gambier Is., Agakauitai	Before AD 1834±5yrs	Musée des Ȋles, Tahiti, No catalogue number. Bag A1 [20] and Fig 1C
BQUCHTE014	Gambier Is., Agakauitai	Before AD 1834±5yr	Musée des Ȋles, Tahiti, No catalogue number. Bag A2 [20]
BQUCHTE015	Gambier Is., Agakauitai	Before AD 1834±5yr	Musée des Ȋles, Tahiti, No catalogue number. Bag B2 [20]
BQUCHTE016	Gambier Is., Agakauitai	Before AD 1834±5yr	Musée des Ȋles, Tahiti, No catalogue number. Bag B1 [20]

(?) Indicates uncertain provenance.

*Specimen accession numbers are given when available.

### DNA extraction

In order to assess reproducibility of results, we extracted DNA in triplicate from all textiles whenever possible. Some samples were extracted in duplicate or only once due to their small size. DNA extraction from all samples was performed at the ancient DNA facilities of the University of Otago, following the CTAB protocol described in Seelenfreund *et al*. [20]. For the aDNA extraction, fragments ranging from approximately 10 mg (of approximately 1 cm2), were taken from each textile sample. For each sample set, negative controls were included, which were processed simultaneously. Each sample was torn and cut lengthwise with a scalpel on a clean surface before adding reagents for DNA extraction. DNA extractions were performed according to the protocol designed and described in [16] with some additional modifications informed in [20]. This protocol consisted of a homogenization of the tissue in 1000 μL extraction buffer containing 2% CTAB, incubation at 60°C overnight, extraction of proteins and impurities with organic solvents (chloroform and isoamylic alcohol 24:1) and precipitation of nucleic acids using cold absolute ethanol in the presence of salt (NaCl 5M). Finally, DNA was resuspended in distilled water. Negative controls contained extraction buffer but lacked the study sample were treated exactly as all samples for the rest of the extraction protocol. DNA concentration and purity (Absorbance ratio 260 /280) were measured using a NanoDrop ND-2000 spectrophotometer (NanoDrop Technologies, Wilmington, DE, USA) and by fluorescence analysis using the Quant-iT™ PicoGreen® dsDNA Assay Kit (Life Technologies, Carlsbad, CA, USA) with a quantitation range of 50–2 pg, according to the manufacturer's instructions. Integrity of the extracted DNA was assessed by electrophoresis on 0.8% agarose gels. In order to improve amplification, colored samples were purified using the MinElute PCR Purification Kit (Qiagen, Hilden, Germany) following manufacturer’s instructions. DNA extraction of Gambier Islands samples (in triplicate) was reported in Seelenfreund et al. [20].

Criteria used to evaluate the quality of the DNA obtained were total amount of extracted nucleic acids by spectrophotometry (260 nm absorbance) and fluorimetry (Picogreen assay) to detect double stranded DNA. Purity of extracted DNA was calculated by the 260/280 ratio and integrity of the extracted DNA was determined by electrophoresis on agarose gels.

### Amplification, sequencing and genotyping

Following recommendations for work with ancient DNA [26], two or three independent amplifications (depending on the sample) were performed at three different facilities: i) the ancient DNA facility of the Department of Anatomy, University of Otago, ii) the ancient DNA laboratory of the Faculty of Medicine, University of Chile, and iii) the adapted ancient DNA laboratory at the Dept. of Biochemistry and Molecular Biology, Faculty of Chemical and Pharmaceutical Sciences, University of Chile. In addition, three different DNA polymerases were used. Analyses at the University of Otago were performed with KAPA HiFi Hot Start DNA Polymerase (Kapa Biosystems, Wilmington, MS, USA), while GoTaq® G2 Flexi DNA Polymerase and GoTaq® G2 Hot Start Polymerase (Promega, Madison, WI, USA) were used at the facilities in Chile. To improve the yield of the PCR, Bovine Serum Albumin (BSA) was added to all amplification reactions, as recommended by Rohland and Hofreiter [27]. The amplifications were performed using PCR programs of 40 cycles, with reagents and materials used exclusively for work with ancient DNA, as described by Payacán et al. [24]. Samples were characterized using different molecular markers, in order to compare with contemporary plants and herbaria samples [25]. Starting material for all amplifications was DNA obtained from the protocol extraction described previously. In each case, PCR reactions were carried out using 2 μL of DNA extracted directly from the bark cloth samples. PCR reactions included a negative (blank) control, adding the appropriate amounts of sterile distilled water to the mix in all experiments. We analyzed i) the nuclear ITS-1 region using ITS-A (5’-GGA AGG AGA AGT CGT AAC AAG G-3’) and ITS-C (5’-GCA ATT CAC ACC AAG TAT CGC-3’) primers as described [20, 24] and ii) a set of ten nuclear microsatellite (SSR) markers, as reported previously [28]. DNA from all samples was also assayed with a specific paper mulberry sex marker [23] and described in the Supporting Text. Two samples from contemporary *B*. *papyrifera* leaves from Taiwan (accession numbers BQUCH0137 and BQUCH0140) from our genomic DNA bank were amplificated in a separate laboratory as positive reaction controls.

### Data analysis

#### ITS-1 region

Sequencing of amplicons was performed at Macrogen Inc. (Seoul, South Korea). Electropherograms were checked using Bio Edit 7.1.3.0 software [29] and polymorphisms were determined by sequence alignment using the Clustal W algorithm [30] of the CLC Sequence Viewer software, as described [22, 24]. Species identification was based on the ITS-1 sequence using the BLAST tool available at NCBI data base (https://blast.ncbi.nlm.nih.gov/Blast.cgi). Identity of *B*. *papyrifera* was confirmed by sequences presenting E values close to zero, query cover close to 100% and identities higher than 98% [31].

#### Microsatellites

Amplicons were analyzed by capillary electrophoresis at the sequencing services from the Pontificia Universidad Católica de Chile (Santiago, Chile) in an ABI PRISM 3130xl sequencer using the GeneScan™ 500 TAMRA™ dye Size Standard using a GSLIZ500 ladder. Electropherograms were visualized with Peak ScannerTM v1.0 software (Applied Biosystems). As an additional control, all PCR reactions included amplification of a contemporary sample of known genotype used to calibrate allele sizes. Allele sizes were registered in an Excel spreadsheet, as described [24]. Alleles found in samples in this study were compared with alleles in contemporary paper mulberry and herbaria samples from our collection [25]. In order to ascertain that the new alleles detected in these textile samples did not correspond to capillary electrophoresis artifacts, a control experiment was performed. For this, experimental mixtures of 1μl of DNA from contemporary leaf samples of known genotypes were mixed and amplified. All alleles detected separately in each sample were also identified in all mixtures (S2 Fig). Specific and shared alleles from textile, herbaria or contemporary samples were plotted using SigmaPlot software v.14, San José, CA, USA (www.systatsoftware.com).

## Results

### Evaluation of DNA integrity and quantification

DNA was successfully extracted at least once from each sample of barkcloth. Samples from textiles from New Guinea (BQUCHTE004), the two textiles from Fiji (BQUCHTE007, BQUCHTE008), one from Hawai’i (BQUCHTE011), the textile from American Samoa (BQUCHTE012) and the Gambier Islands textile (BQUCHTE013 to BQUCHTE016) were successfully extracted in triplicate. Other samples from textiles from Hawai’i were extracted in duplicate (BQUCHTE002, BQUCHTE003, BQUCHTE006, BQUCHTE009 and BQUCHTE010) and the samples from the Rapa Nui textile (BQUCHTE001) and Hawai’i (BQUCHTE005) could be extracted only once due to their small size. The sample from New Guinea showed a 260/280 absorbance ratio around the optimal 1.7–2.0 range, as did samples BQUCH0007 from Fiji and BQUCH0011 from Hawaii. All other samples presented 260/280 values of 1.1–1.5, indicating co-extraction of proteins, polysaccharides and other cellular components. The basic criteria for including samples for analysis was amplificability with specific markers (see below), even though values of the 260/280 ratio were outside the optimal range. Quantification using Picogreen did not yield any data, possibly due to the low amount of double stranded DNA. The integrity of the extracted DNA is shown in S3 Fig, where highly degraded DNA can be observed.

### Species identification by genetic analysis of the ribosomal ITS region

**Fig 2:**
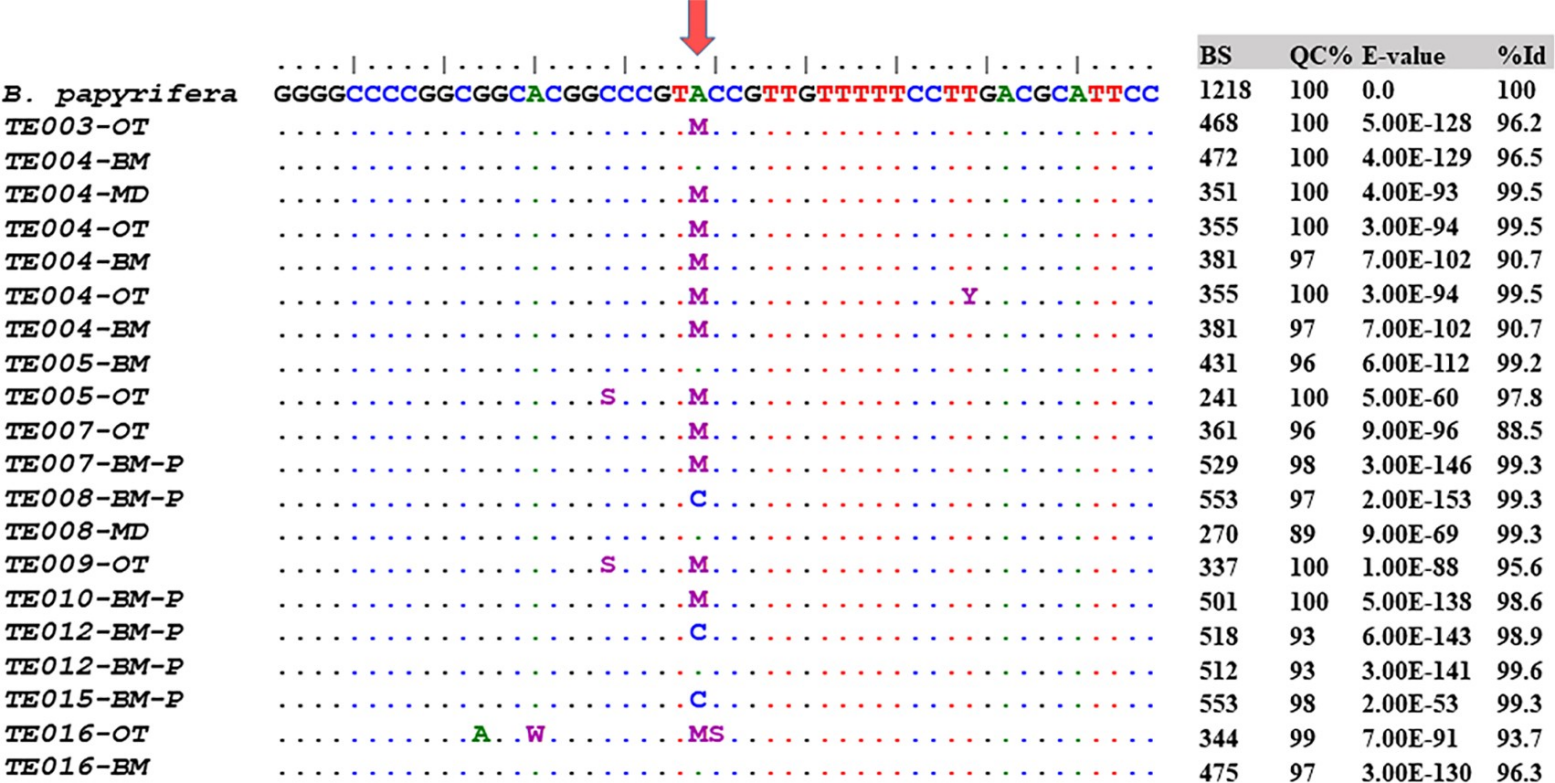
Polymorphisms detected in the ITS-1 sequence of paper mulberry barkcloth textiles. Multiple alignment of 20 selected partial ITS sequences (50 bp) of textile samples. Image adapted from Bioedit 7.1.3.0. The reverse sequence (with primer ITS-C) is shown. The red arrow indicates the polymorphic nucleotide at the relative position 203 [21]. HM623778 corresponds to the ID of the *B*. *papyrifera* ITS region published in Genbank. M = double signal A/C at this position. Y = C or T; S = C or G; W = A or T. Bite Score (BS), Query Coverage (QC), E-values and identity percentage (Id) are indicated on the right. Extension names OT = sample amplified at the University of Otago using KAPA HiFi Hot Start DNA Polymerase; BM = sample amplified using GoTaq® G2 DNA Polymerase at the Faculty of Chemical and Pharmaceutical Sciences, University of Chile; MD = sample amplified using GoTaq® G2 Hot Start Flexi DNA Polymerase at the Faculty of Medicine, University of Chile, and P = sample amplified after extract purification.

From the 16 samples corresponding to 13 different textiles, we prepared 39 DNA extractions, in order to amplify the ITS-1 region. Eleven of these samples yielded 60 amplicons (120 forward and reverse sequences) of independent replicates, of which 106 were readable (Forward and Reverse, or only the Forward or only the Reverse sequence). Edited electropherograms yielded sequences between 155 and 300 base pairs (bp). Of these 106 readable sequences, we identified DNA from various contaminating species in 70 sequences, such as *Triticum aestivum*, *Sporobolus fertilis*, *Helianthus annus*, *Fagus sylvatica*. Thirty-six sequences were identified as *B*. *papyrifera* with statistical significance, corresponding to nine textiles. Specifically, the search for ITS-1 sequences determined that seven textiles, corresponding to samples BQUCHTE004, BQUCHTE005, BQUCHTE007, BQUCHTE008, BQUCHTE010, BQUCHTE012 and BQUCHTE015, were manufactured from paper mulberry bark with an E-value close to zero and > 98% identity (Fig 2). Additionally, samples BQUCHTE003, BQUCHTE009 and BQUCHTE016 presented significant E-values and the characteristic polymorphism at position 203, although identity was less than 98 per cent; these samples were also considered as being made of paper mulberry. At the polymorphic position 203 these samples presented a double peak, suggesting the contribution of two or more genetically different plants. Results for sample BQUCHTE014 did not allow species identification. However, this sample was taken from the same textile as BQUCHTE015 and BQUCHTE016, identified as *B*. *papyrifera*.

Fig 2 shows the sequences that allowed identification of both Pacific and Asian variants based on the characteristic polymorphic site at the relative position 203, as previously described [20, 21, 22]. Of the nine textiles identified as being made of paper mulberry fiber, seven presented both variants (A/C or G/T), as indicated by the presence of a double peak (M) in both forward and reverse sequences, or either in the forward or the reverse sequences. In some cases, different amplifications of a single sample yielded both polymorphisms in separate sequences. For example, the barkcloth sample from Fiji BQUCHTE008 presented both the G variant and the T variant on different amplification reactions. The barkcloth sample from American Samoa (BQUCHTE012) also presented each variant in DNA from different extractions of the sample. In the Gambier Islands textile, sample BQUCHTE016 presented the T variant, while in the BQUCHTE015 sample the G variant was obtained [see S1 Table and 20]. Electropherograms of these samples are shown in S4 Fig.

### Analysis of microsatellite markers

All DNA replicates obtained from the barkcloth samples were analyzed using ten microsatellite markers, as described in the Methods section. The selected markers are those that proved to be most informative for this species, as reported previously [24, 25, 28]. DNA from the New Guinea barkcloth sample amplified with all markers, while DNA from the sample from the Fijian textile amplified four to five markers, and the samples from the barkcloth from the Gambier Islands amplified between six and eight markers. DNA from the Hawaiian sample BQUCHTE010 amplified with three markers and DNA from the American Samoan barkcloth sample amplified with only a single marker. The samples from the Rapa Nui textile and the other six samples from Hawaiian pieces did not produce any amplified DNA with any of the microsatellite markers (Table 2). Amplification success varied depending on the DNA extraction and polymerase used, as summarized in S2 Table.

**Table 2:** Summary of amplification of DNA from barkcloth textile samples with different molecular markers.

Sample code	Locality of origin	ITS-1	Microsatellites
BQUCHTE001	Rapa Nui (?)	Yes	N.A.
BQUCHTE002	Hawaii	Yes	N.A
BQUCHTE003	Hawaii (?)	Yes	N.A
BQUCHTE004	New Guinea	Yes	10
BQUCHTE005	Hawaii	Yes	N.A.
BQUCHTE006	Hawaii	Yes	N.A.
BQUCHTE007	Fiji	Yes	5
BQUCHTE008	Fiji	Yes	4
BQUCHTE009	Hawaii (?)	Yes	N.A.
BQUCHTE010	Hawaii (?)	Yes	3
BQUCHTE011	Hawaii (?)	Yes	N.A.
BQUCHTE012	American Samoa	Yes	1
BQUCHTE013	Gambier Is., Agakauitai	Yes	6
BQUCHTE014	Gambier Is., Agakauitai	Yes	7
BQUCHTE015	Gambier Is., Agakauitai	Yes	8
BQUCHTE016	Gambier Is., Agakauitai	Yes	6

N.A.: No amplification

Table 3 shows alleles detected with each microsatellite marker. Success rate of microsatellite amplification varied for the different textiles, ranging from one to nine samples. Most markers detected more than two alleles in a single amplification reaction: Marker Bro 15 detected five alleles in one sample, while three markers (Bro 08, Bro 15 and Bropap 25444) detected four alleles in six samples. Also, six markers presented three alleles in 14 samples. For example, electropherograms obtained with Bro 13 of samples that present one, two and three alleles are shown in S5 Fig. Only three markers (Bro 07, Bropap 20558 and Bropap 30248) detected one or two alleles per sample.

**Table 3:** Summary of alleles found *per* marker and sample.

SSR marker	Sample	Alleles detected					Total alleles per sample	New alleles per sample	Total alleles per marker	New Alleles per marker
**Bro 07**	BQUCHTE004	**228**	**230**				2	-	2	**-**
**Bro 08**	BQUCHTE004	202	**208**	**214**			3	-	10	***4***
	BQUCHTE008	194					1	-		
	BQUCHTE010	*201*	208	*215*			3	*2*		
	BQUCHTE013	188	202	208	210		4	*-*		
	BQUCHTE014	202	208	*213*			3	*1*		
	BQUCHTE015	194	*213*				2	*1*		
	BQUCHTE016	*201*	202	*213*	*222*		4	*3*		
**Bro13**	BQUCHTE004	220	**226**	***230***			3	*1*	9	***6***
	BQUCHTE007	*206*					1	*1*		
	BQUCHTE008	222					1	*-*		
	BQUCHTE010	*219*					1	*1*		
	BQUCHTE012	220	226	*230*			3	*1*		
	BQUCHTE013	*205*	*216*				2	*2*		
	BQUCHTE014	*216*					1	*1*		
	BQUCHTE015	222					1	-		
	BQUCHTE016	*208*					2	*2*		
**Bro 15**	BQUCHTE004	**210**	**214**	221			3	*-*	12	***7***
	BQUCHTE013	*207*	221				2	*1*		
	BQUCHTE014	*192*	221	*223*	*232*		4	*3*		
	BQUCHTE015	*192*	*196*	202	222	*226*	5	*3*		
	BQUCHTE016	*192*	213	*231*	*232*		4	*3*		
**Bropap 02214**	BQUCHTE004	**241**	245	**247**	249		4	*-*	9	***2***
	BQUCHTE007	*221*					1	*1*		
	BQUCHTE008	239					1	*-*		
	BQUCHTE014	193	*202*				2	*1*		
	BQUCHTE015	227	239				2	-		
**Bropap 2801**	BQUCHTE004	**149**	***155***	**168**			3	*1*	12	***7***
	BQUCHTE007	147	159				2	*-*		
	BQUCHTE008	147	149	*167*			3	*1*		
	BQUCHTE013	*137*	140	***156***			3	*2*		
	BQUCHTE014	*148*	*156*				2	*2*		
	BQUCHTE015	**140**	147	*156*			3	*1*		
	BQUCHTE016	*144*	*146*	***156***			3	*3*		
**Bropap 20558**	BQUCHTE004	217	221				2	*-*	5	***2***
	BQUCHTE007	219	*222*				2	*1*		
	BQUCHTE016	**219**	*233*				2	*1*		
**Bropap 25444**	BQUCHTE004	173	179	**185**	**188**		4	-	5	***-***
	BQUCHTE013	185	189				2	-		
	BQUCHTE014	179	185	189			3	-		
	BQUCHTE015	173	179	185			3	-		
	BQUCHTE016	179					1	-		
**Bropap 26985**	BQUCHTE004	**177**	***181***	**182**			3	*1*	7	***4***
	BQUCHTE007	*173*					1	*1*		
	BQUCHTE010	177					1	*-*		
	BQUCHTE013	*195*					1	*1*		
	BQUCHTE014	177	184				2	*-*		
	BQUCHTE015	*181*	*203*				2	*2*		
**Bropap 30248**	BQUCHTE004	**93**					1	-	2	**-**
	BQUCHTE015	99					1	-		
								**Total**	**73**	***32***

In bold: alleles found in more than one replicate (extractions and PCR reactions) in the same sample using different enzymes. Underlined: alleles found previously exclusively in herbaria samples in Remote Oceania. In italics and underlined with dots: new alleles found in textiles.

**Fig 3:**
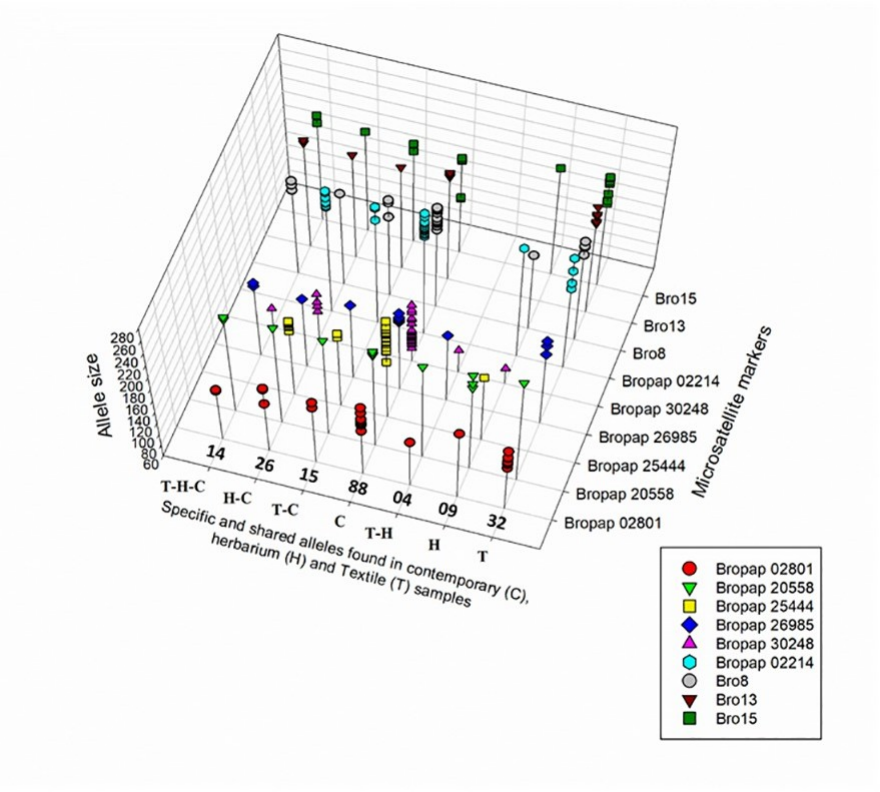
SSR alleles found in textile, herbaria and contemporary paper mulberry samples. 3D scatterplot of alleles found in contemporary plants (C), herbaria (H) and textile samples (T). The X-axis shows alleles that are specific or shared among the different types of samples. The number of alleles is indicated in each case. The Y-axis indicates allele sizes for each marker and sample type. The Z-axis shows the alleles found for each microsatellite marker.

We compared alleles found in the DNA from textile samples with data obtained from contemporary and herbaria *B*. *papyrifera* samples. A total of 73 different alleles were detected; 32 (49%) of these had not been found previously in Oceania, nor in our collection of Asian samples. Markers Bro13, Bro15 and Bropap 2801 presented the highest number of new alleles (Table 3 and Fig 3). We also detected five alleles in textile DNA that have been identified previously in herbaria samples but that were absent in contemporary accessions (Table 3) [25], for markers Bro 08, Bro 15, Bropap 2801 and Bropap 20558. Some samples presented more than two alleles per marker (Table 3 and S5 Fig). In order to ascertain that the new alleles detected in these textile samples did not correspond to capillary electrophoresis artifacts, a control experiment was performed. For this, experimental mixtures of 1μl of DNA from contemporary leaf samples of known genotypes were mixed and amplified. All alleles detected separately in each sample were also identified in all mixtures (S2 Fig). For details on SSR amplification results see S2 and S3 Tables.

### Genetic characterization of three barkcloth textiles

We briefly describe the results for three barkcloth textiles characterized in this work. We selected these three samples because they could be characterized with a largest number of markers.

#### Barkcloth from New Guinea (BQUCHTE004)

**Fig 4:**
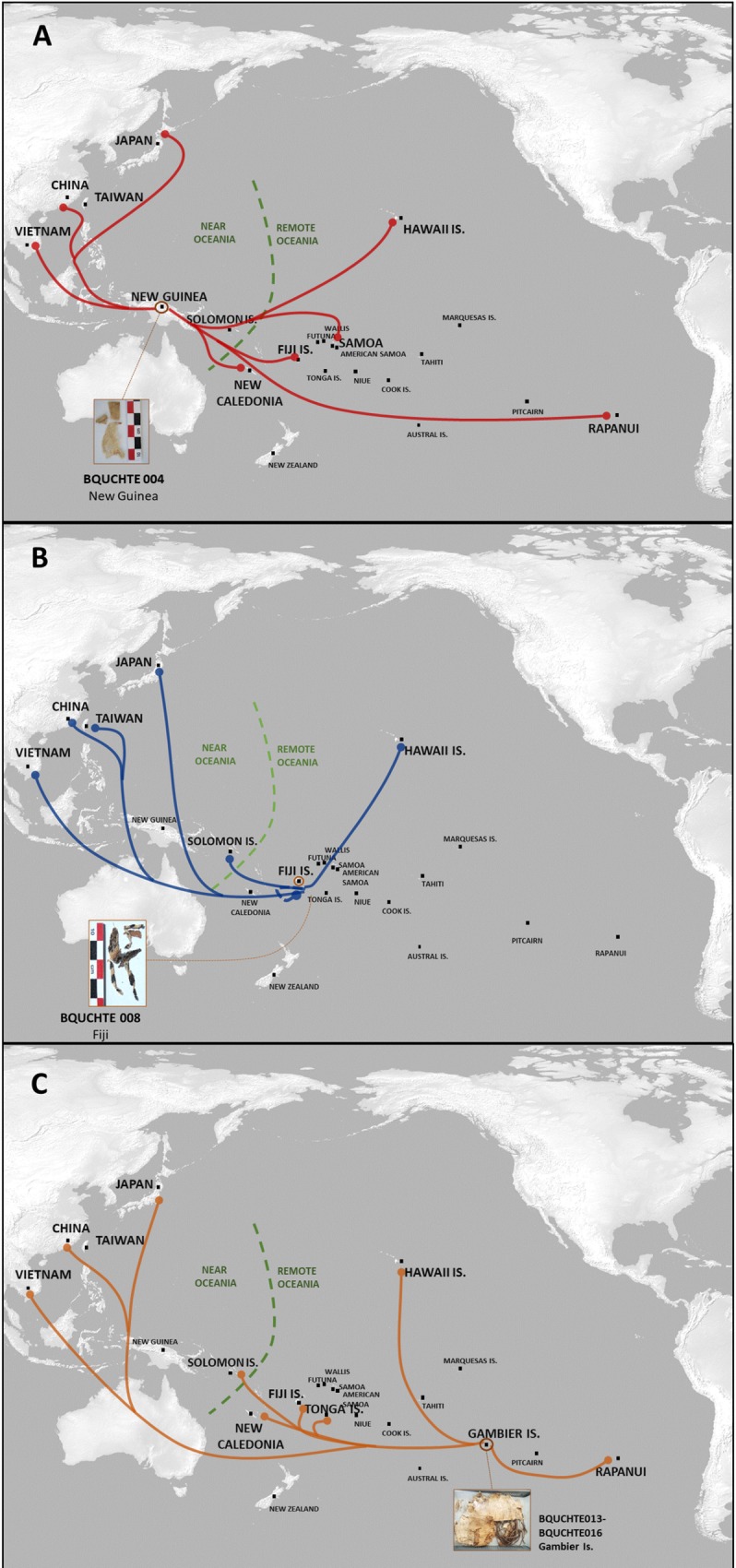
Map of SSR alleles from barkcloth textiles shared with alleles from contemporary and herbarium samples. Stippled line separates Near Oceania from Remote Oceania. (A) Location of alleles detected in the barkcloth textile from New Guinea (sample BQUCHTE004). (B) Location of alleles detected in the barkcloth textile from Fiji (BQUCHTE008). (C) Location of alleles detected in the barkcloth textile from the Gambier Islands (BQUCHTE0013 –BQUCHTE0016). (The base map was made with Natural Earth. Free vector and raster map data @ naturalearthdata.com).

The size of the sample from this textile allowed DNA extraction in triplicate and successful amplification with all tested markers. The ITS-1 sequence identified the fiber source as *B*. *papyrifera* and presented both variants at the relative polymorphic position 203. However, the double-peak signal showed a prevalence of the Polynesian T variant, suggesting that this sample is likely a composite made of bark from at least two or more plants, and each contributing to its genetic makeup. This sample was the only one that could be characterized with the sex marker (see S2 Text and S6 and S7 Figs), determining that this sample was obtained from the bark of female plants. DNA from this sample amplified successfully with all SSR markers. The genetic diversity observed in the SSR analysis indicates the presence of alleles found previously in contemporary and herbaria samples from both Asia and the Pacific: Japan, Taiwan and Vietnam in Asia and in New Caledonia, Fiji, Samoa, Hawaii and Rapa Nui, all part of the introduced range [25]. In DNA from this sample, we detected between one and three alleles per marker, suggesting that two or more genetically different *B*. *papyrifera* plants contributed to the DNA extracted from this textile (Fig 4A and Tables 3 and S3).

#### Barkcloth from Fiji (BQUCHTE008)

We also extracted DNA in triplicate from this Fijian sample. Amplification of the ITS-1 region yielded two sequences identified as *B*. *papyrifera* and, as indicated above, produced the “Pacific” or T variant, and the “Asian” or G variant at the relative polymorphic site 203 (Fig 2). DNA from this sample did not amplify with the sex marker. We successfully extracted and amplified DNA from this sample with four of the ten SSR markers, obtaining one allele not detected previously in herbaria or in contemporary plants from Oceania (Table 3). Results obtained with the ITS-1 region and the SSR markers showed that this textile shares genetic signatures with plants found in the native range in Asia and Hawai’i (Fig 4B).

#### Barkcloth textile from the Gambier Archipelago (samples BQUCHTE013 to BQUCHTE016)

These samples were obtained from an archaeological funerary bundle found in the Gambier Archipelago, French Polynesia, in 2007. Seelenfreund et al. [20] described and genetically characterized these samples using the ITS-1 region. This barkcloth was made of paper mulberry as identified by the ITS-1 marker and presented a double-peak signal at the polymorphic site 203, with the characteristic T variant found in paper mulberry from Remote Oceania samples, as well as the common G variant of Asian plants. Seelenfreund et al. [20] also suggested that several plants were used in the manufacture of this textile (Fig 2). Complementing the initial characterization performed, we further report the analysis of the same samples with ten SSR markers and the sex marker. DNA from three replicate extractions was amplified in duplicate with each marker. These samples could not be characterized with the sex marker assay (see S2 Text). We successfully amplified DNA from this textile with nine SSR markers. DNA from each sample amplified with six to eight microsatellite markers (Table 3). Taken together, the samples BQUCHTE0013, BQUCHTE014, BQUCHTE015 and BQUCHTE016 amplified 45 alleles, of which 24 had not been previously reported. In the case of Bro 08, Bro 13, Bro 15 and Bropap 2801 markers, the new alleles were detected in more than one sample, and in two or more amplifications (for more details per sample, see S3 Table and S2 Text). These samples presented the greatest number of alleles detected to date for any of the analyzed historic barkcloth textiles. We found more than two alleles per marker in five microsatellites, suggesting again that at least two genetically different *B*. *papyrifera* specimens contributed to the DNA extracted from these accessions (see S5 Fig). Of the 19 alleles previously reported, nine had been detected exclusively in Asian samples (China, Taiwan, Japan, and Vietnam), three alleles had been detected exclusively in samples from Remote Oceania and eight alleles were shared by Asian and Remote Oceania populations [25], as shown in Fig 4C. The textile samples from the Gambier archipelago barkcloth presented 23 alleles from seven loci no longer found on any of the islands from Remote Oceania that were sampled.

## Discussion

In this study we report the results of the genetic analysis of historical barkcloth textiles from museum collections and from an archeological context. The selection of these pieces was based on an initial question posed to us by museum conservators relating to the possibility of identifying the species used as a source of fiber to make barkcloth using genetic tools. During this exploratory research new and more complex questions arose, such as the possibility of tracing the origin of unprovenanced samples. This question is important because knowledge of the genetic signature can aid in assigning origin to undecorated tapa pieces of unknown provenance held in various museum collections around the world. Genetic analysis also may provide the tools to reassess doubtful classifications based on design or due to incomplete or missing labels. To address these questions, specific molecular markers had to be selected based on amplicon size (see below); as barkcloth manufacturing involves harsh treatment of the bark fiber, even DNA from modern barkcloth samples is highly degraded [16]. The samples of historical barkcloth in this study were expected to contain highly degraded and low-quality DNA, because of the destructive beating process used during their manufacture. Although in most cases the collection date of the textiles is known, their date of manufacture is unknown. As some of these artifacts may have been saved and inherited for generations, they may be older than the date of registration in the museum or collection.

We report the results of the genetic analyses of tapa samples that yielded amplifiable DNA which indicates that, in each case, these were made from bark from a single plant species—paper mulberry (*B*. *papyrifera*). These samples were further characterized with one to ten microsatellite markers. Finally, we characterized samples from the three textiles that amplified the highest number of markers and results were compared with data from contemporary and herbaria paper mulberry [24, 25]. To our knowledge, this is the first published report of the use of microsatellite markers to analyze DNA from historical barkcloth textiles. These analyses allowed us to detect a genetic diversity not seen previously in herbaria or contemporary samples. However, assignment of un-provenanced textiles to specific islands or archipelagoes remains an unsolved issue.

All samples were extracted following stringent ancient DNA protocols. DNA was highly degraded and fragmented (S3 Fig), and for this reason, it was important to choose molecular markers that amplify short DNA fragments [26]. Species specific markers for *Broussonetia papyrifera* used in this work were microsatellites of 100–250 bp and the ITS-1 region of 370 bp, which is present in multiple copies in the nuclear genome. Amplification depends on the preservation of the DNA in each of the samples and may be influenced by factors such as pigments, differences in the manufacturing process, and the presence of contemporary interfering DNA, humidity and/or temperature during later storage conditions [32]. As we said previously the basic criteria for including samples for analysis was amplificability with these species-specific markers (which allow us to analyze and compare with contemporary and herbaria samples), even if those samples showed a 260/280 absorbance ratio outside of the optimal 1.7–2.0 range.

### Identification of barkcloth fiber source

The bark from several plant species is used in the Pacific as a source for making barkcloth, such as *Artocarpus altilis*, *Ficus* sp. and *Pipturu*s sp. in Hawaii [8]. Barcoding of plant species is generally based on chloroplast DNA (cpDNA) markers. However, the inner bark of paper mulberry used for making barkcloth is a strikingly white tissue that does not contain meristem cells and is devoid of cpDNA [33]. In order to identify the plant species used as a source of fiber for the thirteen textiles, the ITS-1 region was amplified. The ribosomal rDNA sequences that contain the ITS-1 region have been widely used at generic and intrageneric levels in plant phylogenetics because of their universality, simplicity and intragenomic uniformity, among other properties [34]. In spite of the harsh manufacturing process, the use of stringent ancient DNA protocols permitted the recovery of paper mulberry DNA in samples from nine textiles. Identification of paper mulberry was based on the presence of sequences with statistically significant E-values, query cover (sequence coverage) and high percentage of identity, as well as the presence of the characteristic polymorphism.

It is important to keep in mind that tapa textiles are cultural objects made to be used, displayed and gifted within or among communities. They were later given to or collected by European explorers and missionaries and stored without any knowledge or consideration for future genetic analysis. Therefore, they have been subjected to various types of potential contamination through time. Using the universal ITS-1 marker, we detected sequences from species that contaminated the samples at some stage of the life history of each artifact. The risk of modern contaminating species is that their DNA may obscure the scarce endogenous DNA remaining in very old or historic samples. Gilbert el at. [35] suggest that when evaluating DNA from ancient artifacts or of biological origin, consideration should be taken on a case-by-case basis.

In addition, to the identification of the species, the presence of heterogeneity of the plant material used for making the textiles was detected by the presence of both the T and G variants at position 203 in ITS-1 (Fig 2), in spite of using the same protocols as in previous studies. The T variant is characteristic of extant plants in the Pacific, while the G variant is characteristic of Asian plants [21]. The presence of both variants in DNA extracted from a single barkcloth sample, as found in textiles from New Guinea, Fiji and the Gambier Islands, indicates that in the past both variants of this nuclear sequence may have existed in Remote Oceania. In most samples were both variants were detected the signal of the T variant was stronger than of the G variant (S4 Fig). All contemporary plants and herbaria specimens from Remote Oceania present the T variant, except for male plants from Hawaii that present the G variant and Asian microsatellite genotypes known to correspond to recent introductions [25].

### Genetic footprints found in barkcloth samples from the Pacific

Olivares et al. [25], previously showed that microsatellites provide higher resolution for detecting intraspecific genetic diversity than other markers. Analyzing contemporary and herbaria *B*. *papyrifera* samples with ten microsatellites markers, 64 different genotypes were identified in contemporary plants from the Pacific islands; in herbaria samples, additional alleles were found that were not present among contemporary samples of *B*. *papyrifera* in this region. Here we used these same markers to determine if we could identify genetic diversity that allow assignment of un-provenanced textiles to specific islands or archipelagoes. The analysis of the historical textiles in this study yielded five alleles previously recorded exclusively in the herbaria samples and 32 additional alleles that had not been previously identified in either contemporary or herbaria samples (Table 3). However, several of the new alleles are detected in textiles from islands or locations that were under-sampled in the previous studies for various reasons: In some cases, such as New Guinea, our previous data set of contemporary and herbaria specimens included a very limited number of samples and locations within this large island. In the case of Samoa, the extant and herbaria samples are mostly from Western Samoa. Only one sample was provenanced to American Samoa. In general, American Samoa and its surrounding islands are under-represented in the herbaria collections. We do not know if the genetic diversity of paper mulberry from American Samoa differs from the genetic makeup of plants found in Western Samoa. In the case of the Gambier Islands there are no extant plants on the islands today and, as far as we know, no herbaria samples exist in any collection around the world. The closest island that was sampled is Pitcairn that has a very different history. Of the 32 new alleles identified, eight were detected repeatedly. These were observed in DNA amplified from more than one extraction, and /or in two samples from one or more barkcloth textiles; 24 of these new alleles were found only once. These alleles may be suggestive of additional diversity. In the future, it will be of interest to examine if these alleles are present in ethnographic Pacific textiles from other collections.

The presence of three or more microsatellite alleles in a single sample suggests that each barkcloth textile was manufactured from several plants of different genetic backgrounds, as all genetic analyses of extant and herbaria samples presented a single allele (homozygous) or a double signal (heterozygous) for each *locus* [24, 25, 28], as expected in a diploid species. The presence of three or more microsatellite alleles in a single sample is consistent with the presence of ITS variants in a single sample as detected in several textiles and discussed above. The mixture of DNA from different plants precludes the assignment of specific genotypes, but still allowed recording the range of alleles in each analyzed sample. It is no surprise that large barkcloth textiles are a composite of the bark from several plants. Our study shows that it is possible to tease apart the genetic mixture found in very small barkcloth samples. It is of note that the analysis of these minute samples reveals the presence of new alleles in the historical textiles that were not recorded in our collective data set of over 300 contemporary and twentieth century herbaria plant samples from many islands and island groups. In addition to revealing the composite nature of the textiles, these results also suggest the loss of alleles in Pacific paper mulberry plants over the last hundred years.

### Asian and Remote Oceania footprints in historic textiles

Through the alleles detected in each sample, we have been able to reveal connections between the plants used in the past to manufacture these textiles, with contemporary populations of *B*. *papyrifera* from the native range in Asia and the introduced range in the Pacific. The three characterized textiles share alleles from Asia and Remote Oceania, reflecting the human mediated dispersal history of paper mulberry out of mainland Asia, through Taiwan and into Remote Oceania.

The textile sample from the New Guinea barkcloth is the most recent sample characterized, and the only one from which all markers were successfully amplified. The textile originates from Maisin, located on the coast of Collingwood Bay (Oro Province, Papua New Guinea), where people still manufacture and value traditional barkcloth. The presence of alleles from Asia and from Remote Oceania is consistent with the genetic structure found in contemporary Pacific paper mulberry, where plants from New Guinea represent the connecting link between Asia, Near and Remote Oceania [25]. This structure is also consistent with the distribution of mitochondrial DNA (mtDNA) lineages in human populations across Oceania [36]. In a similar way, the sample obtained from a barkcloth textile from Fiji presents several alleles that link this sample to paper mulberry from the native range and to alleles found in female plants from Hawaii.

The allelic diversity found in the samples taken from the barkcloth bundle from the Gambier archipelago is astounding, as 22 alleles no longer detected on any other island in the vast region of Remote Oceania were identified. Their local disappearance highlights the significant loss of Pacific germplasm for this species. Other alleles found in these samples, and previously reported for Remote Oceanic locations, such as Hawaii, New Caledonia, Fiji, Rapanui and Tonga, suggest an early dispersal of the plants from the Gambier Islands. Unexpectedly, the genetic makeup of this unique pre-European contact artifact, encompasses all of the variation found from the source populations in the native range in East Asia and throughout Near and Remote Oceania, and in this way tells and summarizes the dispersal history of paper mulberry from East Asia through the western and eastern regions of Remote Oceania [9, 25]. The presence of numerous alleles exclusive to Taiwan that were also found in this sample, reinforces that paper mulberry accompanied the prehistoric Austronesian voyagers out of Taiwan, all across the Pacific Ocean [9]. Today, paper mulberry is locally extinct in the Gambier Islands [37] and therefore this textile represents the sole evidence of the former diversity of the paper mulberry in this island group. The decline and the final extirpation of this plant in the Gambier archipelago was initiated by the conversion of islanders to Christianity and the progressive loss of traditional culture and substitution of barkcloth by European-made textiles and paper. The European missionaries used barkcloth as a substitute for hard to obtain paper during their initial presence on the island, as attested to by a cutting of white tapa from the covering of the first wedding registry book of the Gambier Mission from 1835, housed in the Museum Te Fare Manama, in Tahiti (catalogue number 474). The loss of paper mulberry plants from this island group sadly reflects the loss of the history and traditions of the peoples that tended these plants.

### Assignment of un-provenanced textiles to specific islands?

In this study we also asked if we might be able to detect genetic connections that had not been previously identified through the analyses of herbaria or contemporary samples. The answer to this question is not straight forward, as the allele diversity found in contemporary and herbaria material is different from the alleles detected in the historic barkcloth textiles. For this reason, the genetic characteristics found in modern or herbaria material cannot be used as a reference for the historical textiles analyzed. This result was surprising, because the alleles found previously were expected to represent a substantial part of the genetic diversity of paper mulberry, a crop that is propagated clonally in Oceanian islands. This limited range of alleles is in some ways analogous to the loss of diversity observed in other canoe species such as *Rattus exulans* [38]. In this regard, we can draw parallels with the genetic diversity found in extant Pacific rats, which is not the same as the diversity of past rat populations in the same geographical areas. This also applies to human populations, as the current inhabitants of some Pacific islands are genetically different than the earliest inhabitants in the same localities [39]. In order to determine the provenance of barkcloth textiles, it will be necessary to analyze a larger number of historical textiles of known origin as reference material, highlighting the value of the barkcloth textile collections housed in museums in different parts of the world. Even with our extensive database of contemporary and herbaria samples we missed part of the diversity of past Pacific paper mulberry plants. The analysis of textile collections of known origin may provide evidence of greater genetic diversity that could potentially allow us built a data base of the past genetic allele diversity. This knowledge in turn may allow identification of specific source populations within the Pacific and therefore determine provenance of particular textiles.

Historic barkcloth textiles can be added to other cultural materials of biological origin from archival or archaeological contexts, such as parchment and papyri, amenable to genetic analysis to reveal past human history and to open windows to the past [40].

## Supporting information

S1 Fig Barkcloth textile samples included in this study. (TIF)

S2 Fig Electropherograms of SSR marker Bro15: Amplification of experimental mixtures of genomic DNA from three contemporary leaf samples. A-C. Electropherograms of individual leaf samples, BQUCH0164, BQUCH0166, and BQUCH0497, respectively. D. Electropherogram of experimental mixing of genomic DNA. Alleles present in all samples are indicated with arrows: BQUCH0164, blue and red arrows; BQUCH0166, green arrow; BQUCH0497, orange arrow. Y-axis shows fluorescence units and X-axis represents DNA fragment size in bp. Size in bp is framed in green.
(TIF)

S3 Fig Integrity of DNA obtained from textile samples. Electrophoresis on 0.8% agarose gel. Sample BQUCH140 corresponds to DNA obtained from contemporary leaf specimen. E.C. = Extraction Control.
(TIF)

S4 Fig Selected ITS-1 sequences obtained from barkcloth samples. All electropherograms correspond to the reverse sequence obtained with ITS-C primers. The arrow indicates the polymorphic nucleotide at the relative position 203 [21].
(TIF)

S5 Fig Electropherograms of six samples from five textiles amplified with the Bro 13 SSR marker. Images adapted from electropherograms shown by the Peak Scanner v1.0 program. The identification code, locality of origin and identified genotype of each sample in the corresponding electropherogram is indicated. The box indicates allele sizes (peaks shown correspond to raw data, peaks sizes include the M13 tail). Sample BQUCH0137 corresponds to a contemporary leaf sample (control).
(TIF)

S6 Fig New primers designed for amplification of the sex marker adapted for DNA extracted from barkcloth. A. Primers designed by Peñailillo et al., [23] to determine sex of *B*. *papyrifera* individuals. B. New primers designed to amplify smaller fragments of the same genomic region.
(TIF)

S7 Fig Sex marker analysis. The female positive control from contemporary leaf sample BQUCH140 presents a single band at 273 pb, while the male positive control (sample BQUCH137) presents two bands at 273 pb and 165 pb. The single sample that presented amplification corresponds to the sample from the textile from New Guinea and was identified as from female plants. E.C: Extraction Control; Water: negative amplification control.
(TIF)

S1 Table DNA samples identified using ITS-1 marker. (DOCX)

S2 Table Summary of microsatellite amplifications indicating samples assayed, facilities and enzymes used and number of positive amplifications for each SSR marker per sample. (DOCX)

S3 Table Alleles obtained in each amplification of SSR and using different polymerases for each facility. (DOCX)

S1 Text Barkcloth descriptions. (DOCX)

S2 Text Redesign and amplification of the sex marker region. (DOCX)

S3 Text Permits. (DOCX)

## References

[1] Matisoo-Smith EA. The Commensal Model for Human Settlement of the Pacific 10 Years on—What Can We Say and Where to Now? Journal of Island & Coastal Archaeology 2009; 4:151–163.

[2] - Falush D., Wirth T., Linz B., Pritchard J. K., Stephens M., Kidd, et al Traces of human migrations in Helicobacter pylori populations. Science; 2003 299;5612: 1582–1585. 10.1126/science.1080857 12624269

[3] - Kirch PV, On the Road of the Winds. An Archaeological History of the Pacific Islands before European Contact. 1st ed., Berkley, Los Angeles: University of California Press Books; 2000.

[4] - White JP. Fauna: More than just food. In, Proceedings of the Fifth International Conference on Easter Island and the Pacific (C. M. Stevenson, G. Lee, and F. J. Morin, eds.). Los Osos: Bearsville Press; 2001: 37–40.

[5] - Lee T, Burch JB, Coote T, Fontaine B, Gargominy O, Pearce-Kelly P. et al. Prehistoric inter-archipelago trading of Polynesian tree snails leaves a conservation legacy. (2007). Proceedings of the Royal Society B: Biological Sciences, 274(1627), 2907–2914. 10.1098/rspb.2007.1009 PMC3227131 17848368

[6] - Storey A A, Clarke A C, Ladefoged T, Robins J, & Matisoo-Smith E. DNA and Pacific commensal models: Applications, construction, limitations, and future prospects. (2013). The Journal of Island and Coastal Archaeology, 8(1), 37–65.

[7] - Renfrew C. Where Bacteria and Languages Concur. Science. 2009; 323: 467–468. 10.1126/science.1168953 19164735

[8] Charleux M. (ed.) Tapa: d L'Ecorce à L'Etoffe, art millenaire d'Oceanie. Del'Asie du Sud-Est à la Polynèsie Orientale / Tapa: From Tree Back to Cloth: an Ancient Art of Oceania. From Southeast Asia to Eastern Polynesia. Paris, Somogy- TAPA Ed. 2017.

[9] Chang C-S, Liu H-L, Moncada X, Seelenfreund A, Seelenfreund D, Chung K-F. A holistic picture of Austronesian migrations revealed by phylogeography of Pacific paper mulberry. Proc Natl Acad Sci USA. 2015; 112: 13537–13542. 10.1073/pnas.1503205112 26438853 PMC4640734

[10] Mesenhöller P, Lueb O. Made in Oceania. Rautenstrauch-Joest-Museum. Kulturen der Welt, Köln, Germany; 2013.

[11] Marota I, Basile C, Ubaldi M, Rollo F. DNA Decay Rate in Papyri and Human Remains from Egyptian Archaeological Sites. Am J Phys Anthropol. 2002; 117: 310–318. 10.1002/ajpa.10045 11920366

[12] Speirs AK, McConnachie G, Lowe A. Chloroplast DNA from 16th century waterlogged oak in a marine environment: initial steps in sourcing the Mary Rose timbers. Archaeological Science under a microscope: Studies in residue and ancient DNA analysis in honour of Thomas H. Loy. Terra Australis. 2009; 30: 175–189.

[13] Hartnup K, Huynen L, Te Kanawa R, Shepherd LD, Millar CD, Lambert DM. Ancient DNA recovers the origins of Māori feather cloaks. Molec Biol Evol. 2011; 28: 2741–2750. 10.1093/molbev/msr107 21558445

[14] Teasdale MD, Van Doorn NL, Fiddyment S, Webb CC, O'Connor T, Hofreiter M., et al Paging through history: parchment as a reservoir of ancient DNA for next generation sequencing. Phil Trans R Soc B. 2015; 370: 20130379 10.1098/rstb.2013.0379 25487331 PMC4275887

[15] Bastian F, Jacot-des-Combes C, Hänni C, Perrier M. Determination of the geographical origin of leather shields from Zanzibar using ancient DNA tools. J Archaeol Sci. Reports 2018; 19: 323–333.

[16] Moncada X, Payacán X, Arriaza F, Lobos S, Seelenfreund D, Seelenfreund A. DNA extraction and amplification from contemporary Polynesian Barkcloth. PLoS One 2013; 8: e56549 10.1371/journal.pone.0056549 23437166 PMC3578839

[17] Kooijman S. Tapa in Polynesia. Bernice P. Bishop Museum Bulletin 234. Honolulu, Bishop Museum Press, 1972, 498 pp.

[18] Neich R, Pendergrast M. Pacific Tapa. University of Hawaii Press; 2004.

[19] Daniels V. The characteristics of modern and old barkcloth (tapa). The Conservator 2005; 29: 95–10.

[20] Seelenfreund A, Sepúlveda M, Petchey F, Peña-Ahumada B, Payacán C, Gutiérrez S, et al Characterization of an archaeological decorated barkcloth from Agakauitai Island, Gambier archipelago, French Polynesia. J Archaeol Sci. 2016; 76: 56–69.

[21] Seelenfreund D, Piña R, Ho KY, Lobos S, Moncada X, Seelenfreund A. Molecular analysis of Broussonetia papyrifera (L.) Vent. (Magnoliophyta: Urticales) from the Pacific, based on ribosomal sequences of nuclear DNA. New Zealand J Bot. 2011; 49: 413–420.

[22] González-Lorca J, Rivera-Hutinel A, Moncada X, Lobos S, Seelenfreund D, Seelenfreund A. Ancient and modern introduction of Broussonetia papyrifera ([L.] Vent.; Moraceae) into the Pacific: Genetic, geographical and historical evidence. New Zealand J Bot. 2015; 53: 75–89.

[23] Peñailillo J, Olivares G, Moncada X, Payacán C, Chang C-S, Chung K-F, et al Sex distribution of paper mulberry (Broussonetia papyrifera) in the Pacific. PloS One 2016; 11: e0161148 10.1371/journal.pone.0161148 27529483 PMC4986985

[24] Payacán C, Moncada X, Rojas G, Clarke A, Chung K-F, Allaby R, et al Phylogeography of herbarium specimens of asexually propagated paper mulberry [Broussonetia papyrifera (L.) L’Hér. ex Vent. (Moraceae)] reveals genetic diversity across the Pacific. Ann Bot. 2017; 120: 387–404. 10.1093/aob/mcx062 28633358 PMC5591419

[25] Olivares G, Peña-Ahumada B, Peñailillo J, Payacán C, Moncada X, Saldarriaga-Córdoba M, et al Human mediated translocation of Pacific paper mulberry [Broussonetia papyrifera (L.) L’Hér. ex Vent. (Moraceae)]: genetic evidence of dispersal routes in Remote Oceania. Plos One 2019; 14(6): e0217107 10.1371/journal.pone.0217107 31216291 PMC6583976

[26] Cooper A, Poinar HN. Ancient DNA: do it right or not at all. Science. 2000; 289: 1139–1139. 10.1126/science.289.5482.1139b 10970224

[27] Rohland N, Hofreiter M. Comparison and optimization of ancient DNA extraction. Biotechniques. 2007; 42(3): 343 10.2144/000112383 17390541

[28] Peñailillo J, Kuo W, Olivares G, Silva-Poblete G, Peña-Ahumada B, Muñoz S, et al Characterization of microsatellite markers for Broussonetia papyrifera (Moraceae). Applications in Plant Sciences 2017; 5(8): 1700044 10.3732/apps.1700044 28924515 PMC5584819

[29] Hall TA. BioEdit: a user-friendly biological sequence alignment editor and analysis program for Windows 95/98/NT. Nucl Acids Symp Series. 1999; 4: 95–98.

[30] Thompson J, Higgins DG, Gibson TJ. CLUSTAL W: improving the sensitivity of progressive multiple sequence alignment through sequence weighting, position-specific gap penalties and weight matrix. Nucl Acids Res. 1994; 22: 4673–4680. 10.1093/nar/22.22.4673 7984417 PMC308517

[31] Pearson WR. An introduction to sequence similarity (“homology”) searching. Curr Protoc Bioinformatics. 2013; 3: Unit 3 1 10.1002/0471250953.bi0301s42 23749753 PMC3820096

[32] Dabney J, Meyer M, Pääbo S. Ancient DNA damage. Cold Spring Harb Perspect Biol. 2013; 5(7): a012567 10.1101/cshperspect.a012567 23729639 PMC3685887

[33] Kress W J, Wurdack K J, Zimmer EA,. Weigt LA, Janzen DH. Use of DNA barcodes to identify flowering plants. PNAS. 2005; 102(23): 8369–8374. 10.1073/pnas.0503123102 15928076 PMC1142120

[34] Álvarez I, Wendel JF. Ribosomal ITS sequences and plant phylogenetic inference. Molec Phylogenet Evol. 2003; 29: 417–434. 10.1016/s1055-7903(03)00208-2 14615184

[35] Gilbert MTP, Bandelt HJ, Hofreiter M, Barnes I. Assessing ancient DNA studies. Ecol Evol. 2005; 30: 541–544. 10.1016/j.tree.2005.07.005 16701432

[36] Kayser M. The Human Genetic History of Oceania: Near and Remote Views of Dispersal. Curr Biol. 2010; 20: R194–R201. 10.1016/j.cub.2009.12.004 20178767

[37] Seelenfreund D, Clarke AC, Oyanedel N, Piña R, Lobos S, Matisoo-Smith EA, et al Paper mulberry (Broussonetia papyrifera) as a commensal model for human mobility in Oceania: anthropological, botanical and genetic considerations', New Zealand Journal of Botany, 2010; 48: 3 10.1080/0028825X.2010.520323

[38] Matisoo-Smith EA. Something Old, Something New: Do Genetic Studies of Contemporary Populations Reliably Represent Prehistoric Populations of Pacific *Rattus exulans*? Human Biology 2002; 74(3):489–96. 10.1353/hub.2002.0032 12180768

[39] Lipson M, Skoglund P, Spriggs M, Valentin F, Bedford S, Shing R, et al Population Turnover in Remote Oceania Shortly after Initial Settlement. Curr Biol. 2018; 28(7):1157-1165.e7. 10.1016/j.cub.2018.02.051 29501328 PMC5882562

[40] Green EJ, Speller CF. Novel substrates as sources of Ancient DNA: Prospects and Hurdles. Genes 2017; 8: 180. 10.3390/genes8070180 PMC5541313 28703741

