# Supplementary figures and images for: A tale of textiles: Genetic characterization of historical paper mulberry barkcloth from Oceania
Source: PLoS One. 2020 May 18;15(5):e0233113. doi: 10.1371/journal.pone.0233113 (PMC7233582; doi:10.1371/journal.pone.0233113)

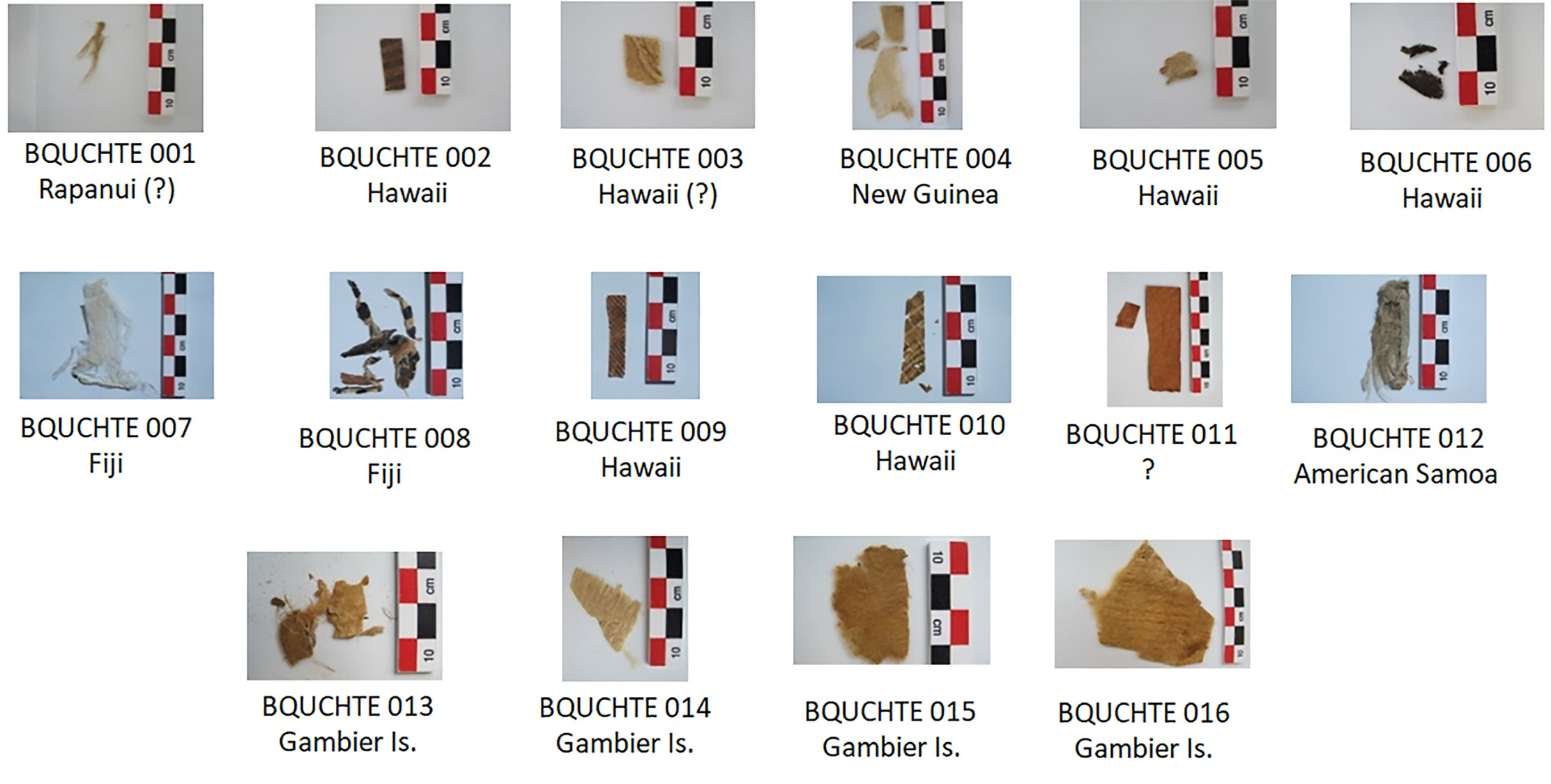

Supplement: S1 Fig — (TIF) [file pone.0233113.s001.tif]

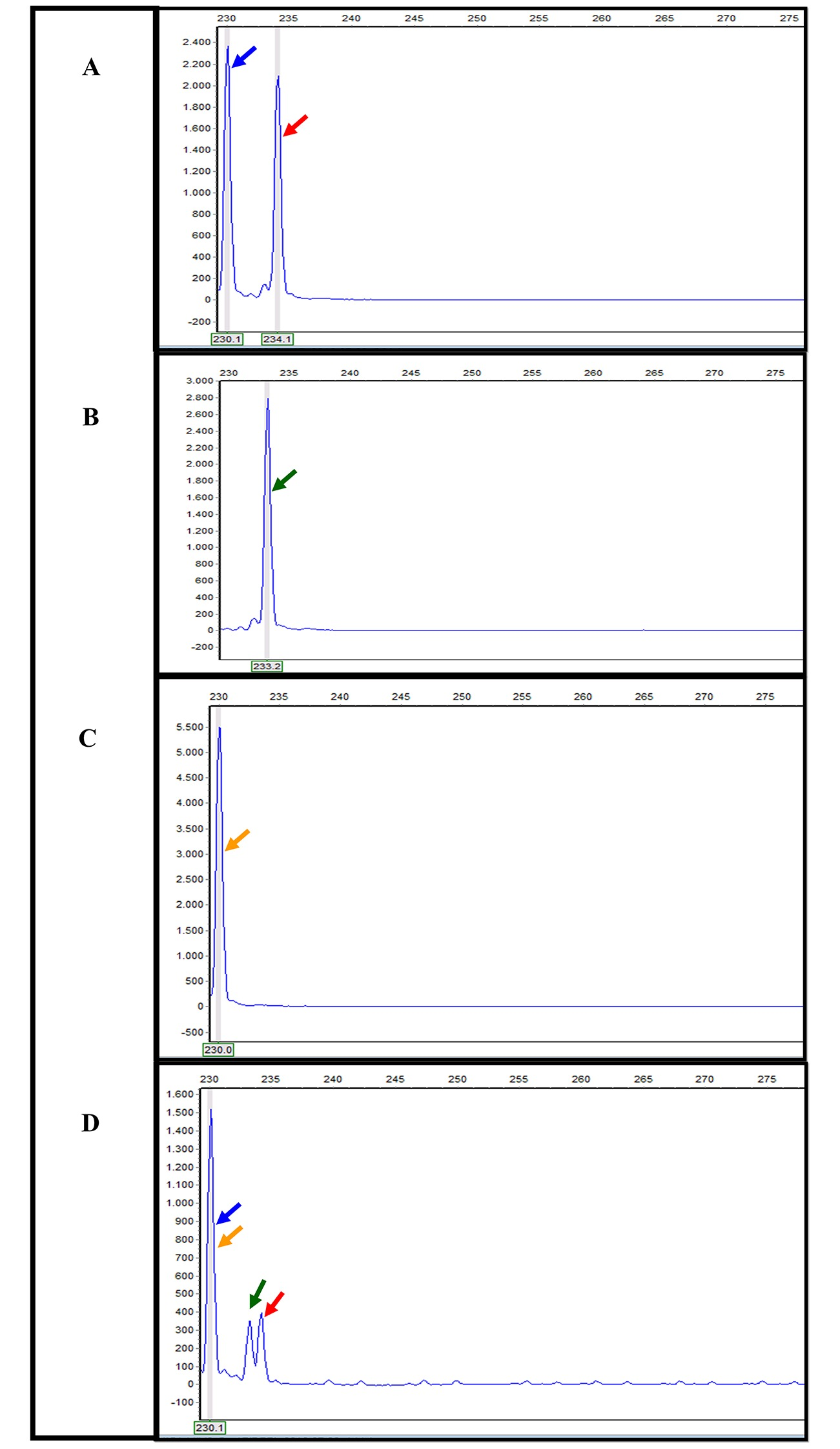

Supplement: S2 Fig — A-C. Electropherograms of individual leaf samples, BQUCH0164, BQUCH0166, and BQUCH0497, respectively. D. Electropherogram of experimental mixing of genomic DNA. Alleles present in all samples are indicated with arrows: BQUCH0164, blue and red arrows; BQUCH0166, green arrow; BQUCH0497, orange arrow. Y-axis shows fluorescence units and X-axis represents DNA fragment size in bp. Size in bp is framed in green. (TIF) [file pone.0233113.s002.tif]

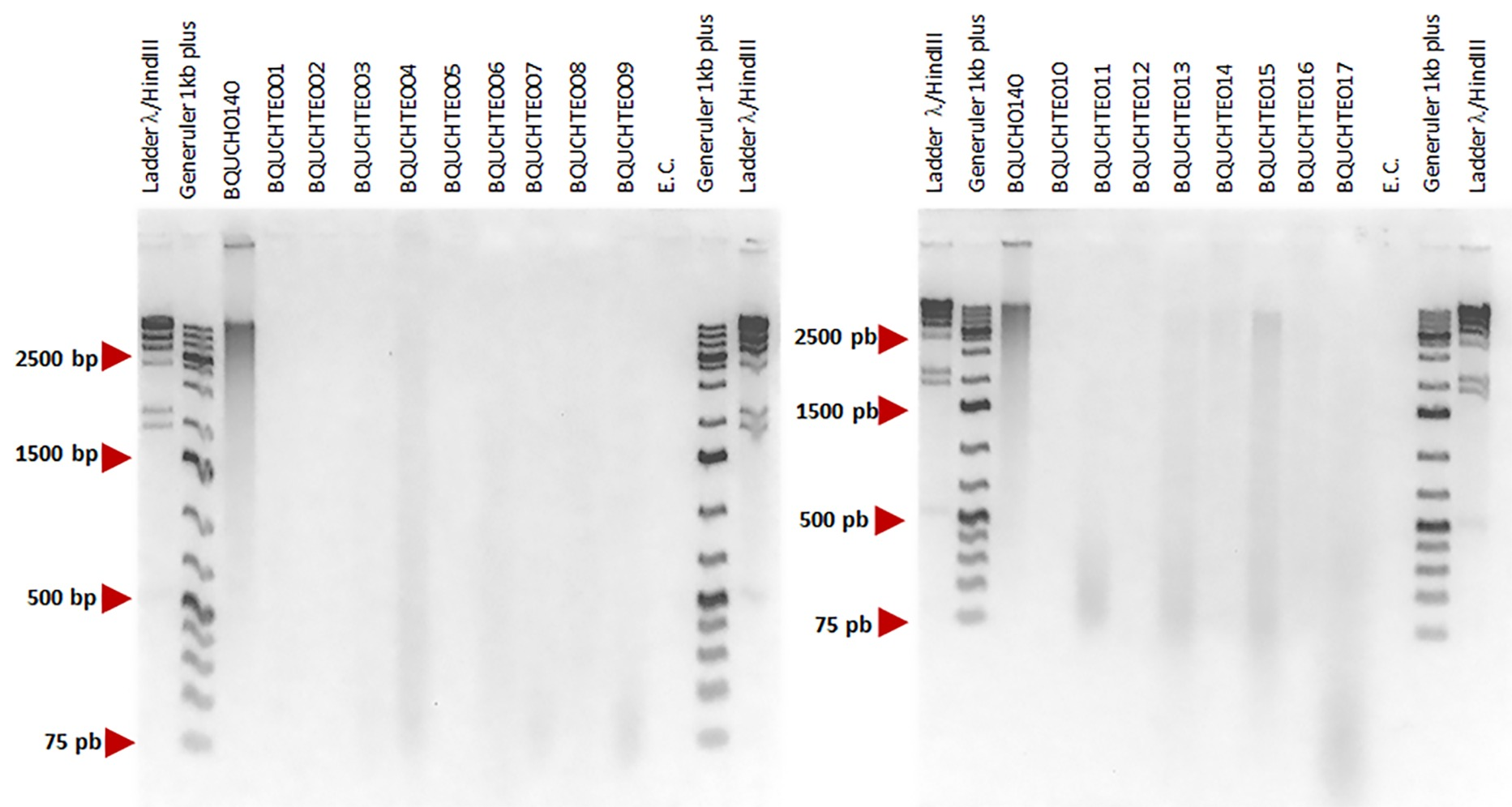

Supplement: S3 Fig — Electrophoresis on 0.8% agarose gel. Sample BQUCH140 corresponds to DNA obtained from contemporary leaf specimen. E.C. = Extraction Control. (TIF) [file pone.0233113.s003.tif]

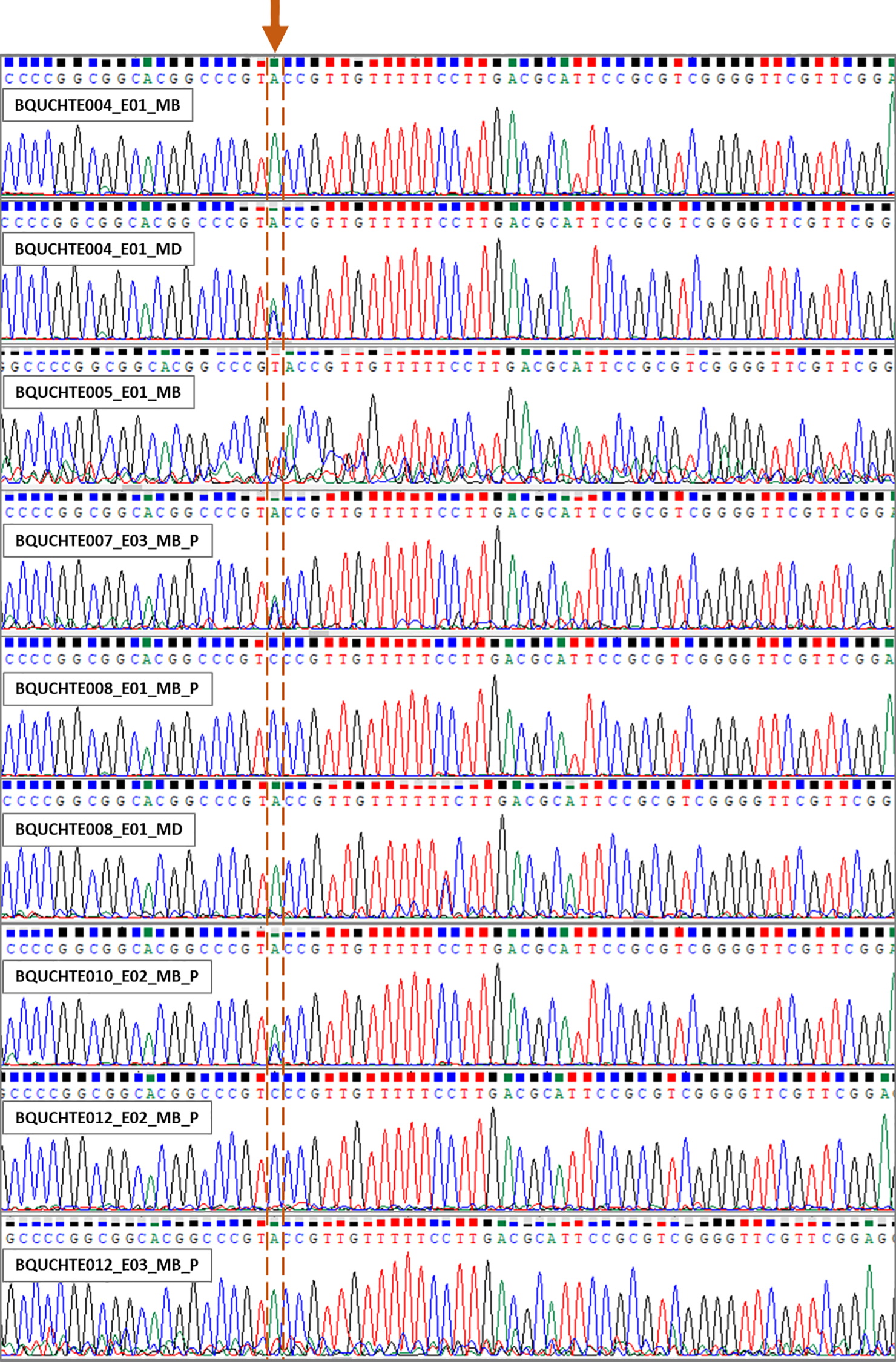

Supplement: S4 Fig — All electropherograms correspond to the reverse sequence obtained with ITS-C primers. The arrow indicates the polymorphic nucleotide at the relative position 203 [21]. (TIF) [file pone.0233113.s004.tif]

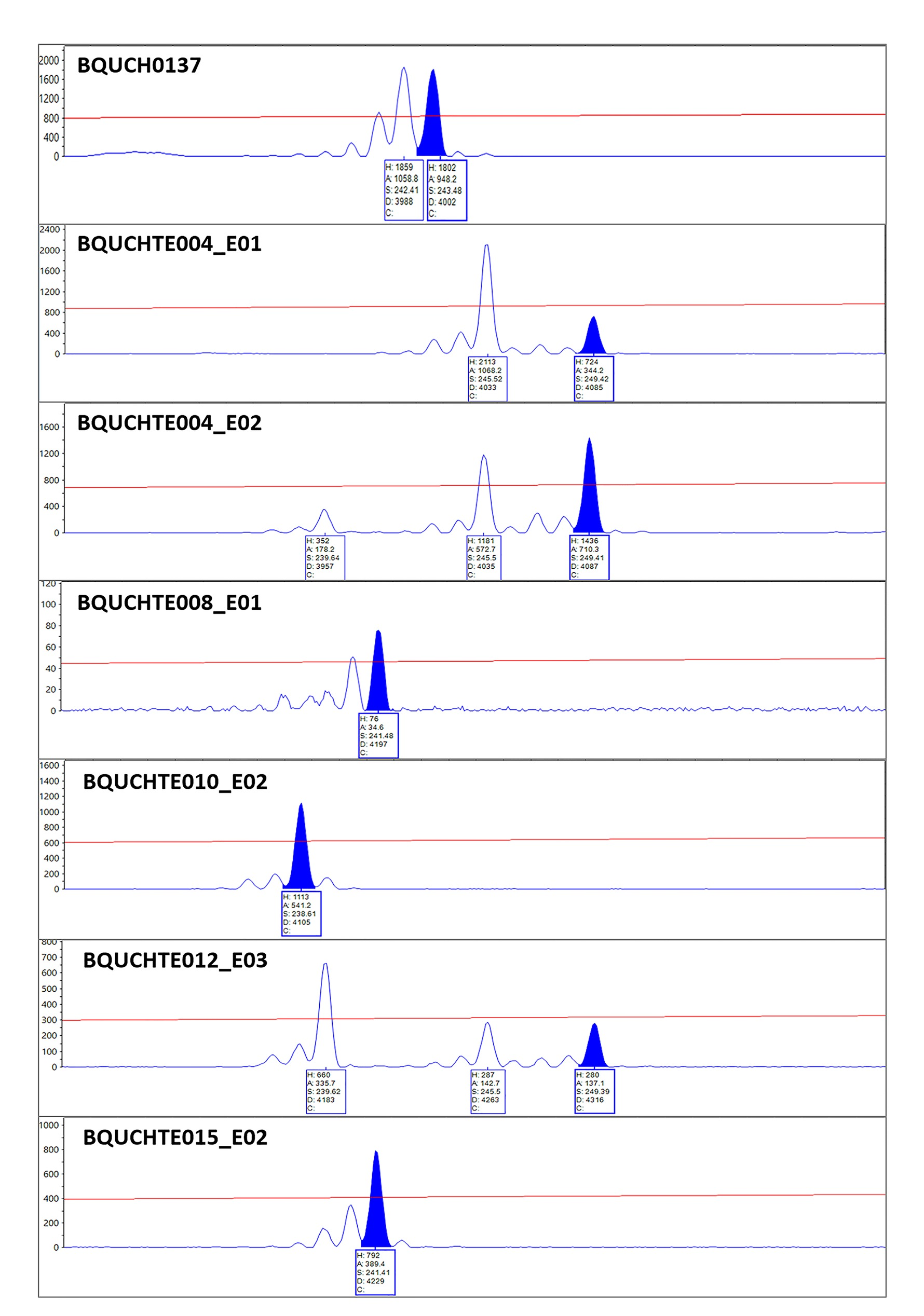

Supplement: S5 Fig — Images adapted from electropherograms shown by the Peak Scanner v1.0 program. The identification code, locality of origin and identified genotype of each sample in the corresponding electropherogram is indicated. The box indicates allele sizes (peaks shown correspond to raw data, peaks sizes include the M13 tail). Sample BQUCH0137 corresponds to a contemporary leaf sample (control). (TIF) [file pone.0233113.s005.tif]

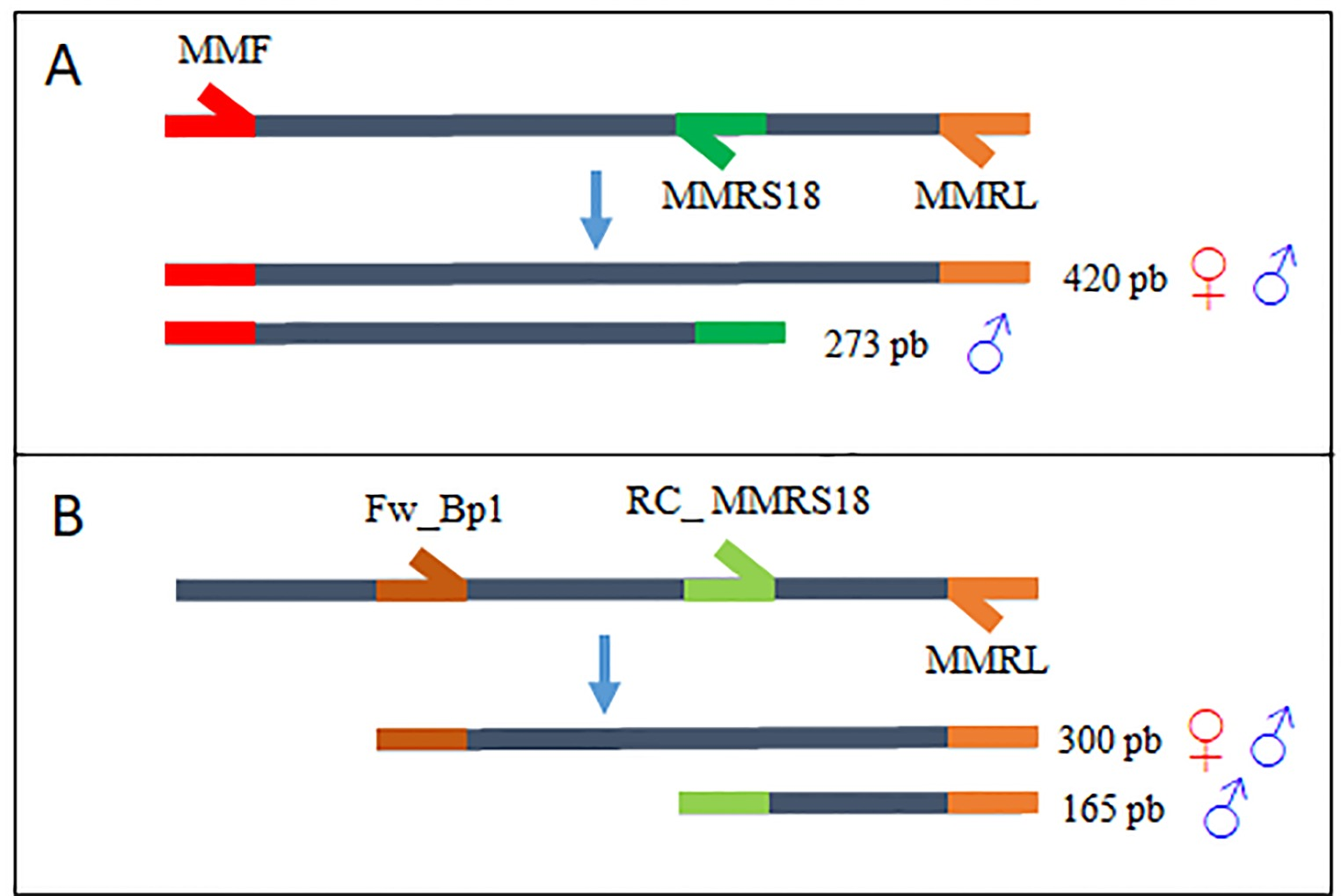

Supplement: S6 Fig — A. Primers designed by Peñailillo et al., [23] to determine sex of B. papyrifera individuals. B. New primers designed to amplify smaller fragments of the same genomic region. (TIF) [file pone.0233113.s006.tif]

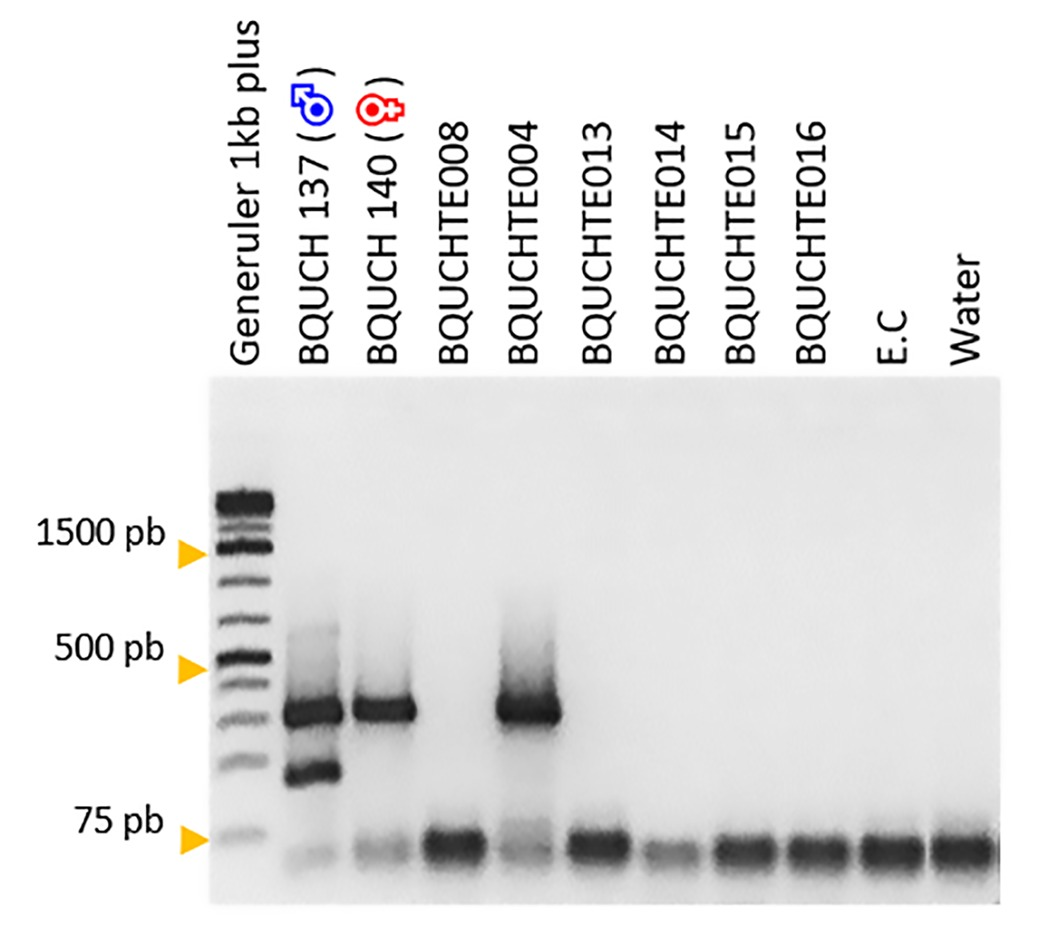

Supplement: S7 Fig — The female positive control from contemporary leaf sample BQUCH140 presents a single band at 273 pb, while the male positive control (sample BQUCH137) presents two bands at 273 pb and 165 pb. The single sample that presented amplification corresponds to the sample from the textile from New Guinea and was identified as from female plants. E.C: Extraction Control; Water: negative amplification control. (TIF) [file pone.0233113.s007.tif]
