# Supplementary material for: A tale of textiles: Genetic characterization of historical paper mulberry barkcloth from Oceania
Source: PLoS One. 2020 May 18;15(5):e0233113. doi: 10.1371/journal.pone.0233113 (PMC7233582; doi:10.1371/journal.pone.0233113)
Supplement: S1 Table — (DOCX) [file pone.0233113.s008.docx]

**S1 Table**. **DNA samples identified using ITS-1 marker.**

| **Sample** | **Sequence** | **Species identified** | **E value** | **% identity** | **Polymorphism** |
| --- | --- | --- | --- | --- | --- |
| BQUCHTE003_E02_OT | ITS-A | *B. papyrifera* | 5.00E-17 | 100 | M |
|  | ITS-C | *B. papyrifera* | 9.00E-66 | 100 | M |
| BQUCHTE004_E01_MB | ITS-A | *B. papyrifera* | 2.00E-115 | 95 | M |
|  | ITS-C | *B. papyrifera* | 3.00E-81 | 100 | A |
| BQUCHTE004_E01_MD | ITS-A | *B. papyrifera* | 6.00E-56 | 80 | M |
|  | ITS-C | *B. papyrifera* | 5.00E-102 | 89 | M |
| BQUCHTE004_E01_OT | ITS-A | *B. papyrifera* | 3.00E-23 | 75 | M |
|  | ITS-C | *B. papyrifera* | 1.00E-80 | 99 | M |
| BQUCHTE004_E02_MB | ITS-A | *B. papyrifera* | 1.00E-45 | 86 | - |
|  | ITS-C | *B. papyrifera* | 7.00E-78 | 98 | M |
| BQUCHTE004_E02_OT | ITS-A | *B. papyrifera* | 1.00E-16 | 87 | M |
|  | ITS-C | *B. papyrifera* | 4.00E-75 | 99 | M |
| BQUCHTE004_E03_MB | ITS-A | *B. papyrifera* | 6.00E-39 | 84 | - |
|  | ITS-C | *B. papyrifera* | 3.00E-81 | 100 | M |
| BQUCHTE005_E01_MB | ITS-A | *B. papyrifera* | 1.00E-80 | 100 | - |
|  | ITS-C | *B. papyrifera* | 4.00E-112 | 99 | A |
| BQUCHTE005_E01_OT | ITS-A | *Unreadable* | 1.00E-16 | 71 | - |
|  | ITS-C | *B. papyrifera* | 9.00E-75 | 96 | M |
| BQUCHTE007_E02_OT | ITS-A | *B. papyrifera* | 7.00E-15 | 100 | - |
|  | ITS-C | *B. papyrifera* | 3.00E-71 | 100 | M |
| BQUCHTE007_E03_MB | ITS-A | *B. papyrifera* | 8.00E-134 | 99 | T |
|  | ITS-C | *B. papyrifera* | 2.00E-120 | 99 | - |
| BQUCHTE007_E03_MB_P | ITS-A | *B. papyrifera* | 3.00E-149 | 100 | - |
|  | ITS-C | *B. papyrifera* | 1.00E-111 | 100 | M |
| BQUCHTE008_E01_MB_P | ITS-A | *B. papyrifera* | 9.00E-144 | 100 | G |
|  | ITS-C | *B. papyrifera* | 7.00E-140 | 99 | C |
| BQUCHTE008_E01_MD | ITS-A | *B. papyrifera* | 5.00E-73 | 100 | T |
|  | ITS-C | *B. papyrifera* | 6.00E-67 | 99 | A |
| BQUCHTE009_E01_OT | ITS-A | *B. papyrifera* | 4.00E-33 | 92 | - |
|  | ITS-C | *B. papyrifera* | 5.00E-74 | 96 | - |
| BQUCHTE010_E01_OT | ITS-A | *B. papyrifera* | 3.00E-138 | 100 | - |
|  | ITS-C | *B. papyrifera* | 9.00E-144 | 99 | - |
| BQUCHTE010_E02_MB_P | ITS-A | *B. papyrifera* | 5.00E-121 | 97 | - |
|  | ITS-C | *B. papyrifera* | 7.00E-83 | 100 | M |
| BQUCHTE012_E02_MB_P | ITS-A | *B. papyrifera* | 1.00E-136 | 99 | G |
|  | ITS-C | *B. papyrifera* | 1.00E-141 | 99 | C |
| BQUCHTE012_E03_MB_P | ITS-A | *B. papyrifera* | 1.00E-48 | 100 | - |
|  | ITS-C | *B. papyrifera* | 6.00E-136 | 99 | A |
| BQUCHTE014_E01_OT | ITS-A | unreadable | - | - | - |
|  | ITS-C | *B. papyrifera* | 2.00E-125 | 99 | - |
| BQUCHTE015_E02_MB_P | ITS-A | *B. papyrifera* | 4.00E-127 | 99 | G |
|  | ITS-C | *B. papyrifera* | 7.00E-140 | 99 | C |
| BQUCHTE016_E01_OT | ITS-A | Unreadable | - | - | - |
|  | ITS-C | *B. papyrifera* | 2.00E-52 | 96 | M |
| BQUCHTE016_E02_MB | ITS-A | *B. papyrifera* | 2.00E-45 | 100 | - |
|  | ITS-C | *B. papyrifera* | 7.00E-120 | 96 | A |

M= A or C
