# Supplementary material for: A tale of textiles: Genetic characterization of historical paper mulberry barkcloth from Oceania
Source: PLoS One. 2020 May 18;15(5):e0233113. doi: 10.1371/journal.pone.0233113 (PMC7233582; doi:10.1371/journal.pone.0233113)
Supplement: S2 Table — (DOCX) [file pone.0233113.s009.docx]

**S2 Table. Summary of Microsatellite amplifications indicating samples assayed, facilities and enzymes used and number of positive amplifications for each SSR marker per sample.**

| **Location of amplification** | **Faculty of Chemical and Pharmaceutical Sciences, University of Chile** | | | **Faculty of Medicine, University of Chile** |
| --- | --- | --- | --- | --- |
| **Enzyme used** | **GoTaq® G2 Flexi DNA Polymerase** | | | **GoTaq® G2 Hot Start Polymerase** |
| **Sample** | **Amplification 1** | **Amplification 2** | **Amplification 3** | **Amplification 4** |
| BQUCHTE001 | - | - | - | - |
| BQUCHTE002 | N.A | - | - | - |
| BQUCHTE003 | - | - | - | - |
| BQUCHTE004 | 6 SSR | 9 SSR | 2 SSR | 10 SSR |
| BQUCHTE005 | N.A | - | - | - |
| BQUCHTE006 | - | - | - | - |
| BQUCHTE007 | 1 SSR | N.A | 3 SSR | - |
| BQUCHTE008 | N.A | 4 SSR | N.A | N.A |
| BQUCHTE009 | N.A | - | - | - |
| BQUCHTE010 | 1 SSR | 2 SSR | - | - |
| BQUCHTE011 | - | - | - | - |
| BQUCHTE012 | N.A | 1 SSR | N.A | N.A |
| BQUCHTE013 | 3 SSR | - | 3 SSR | 2 SSR |
| BQUCHTE014 | 4 SSR | - | 4 SSR | 3 SSR |
| BQUCHTE015 | N.A | 4 SSR | 2 SSR | 3 SSR |
| BQUCHTE016 | 2 SSR | - | 1 SSR | 4 SSR |

N.A: No amplification. - : not assayed.
