# Supplementary material for: A tale of textiles: Genetic characterization of historical paper mulberry barkcloth from Oceania
Source: PLoS One. 2020 May 18;15(5):e0233113. doi: 10.1371/journal.pone.0233113 (PMC7233582; doi:10.1371/journal.pone.0233113)
Supplement: S3 Table — (DOCX) [file pone.0233113.s010.docx]

**S3 Table. Alleles obtained in each amplification of SSR and using different polymerases for each facility**

|  |  | **TE004** | | | **TE007** | | | **TE008** | | | **TE010** | | | **TE012** | | | **TE013** | | | **TE014** | | | **TE015** | | | **TE016** | | |
| --- | --- | --- | --- | --- | --- | --- | --- | --- | --- | --- | --- | --- | --- | --- | --- | --- | --- | --- | --- | --- | --- | --- | --- | --- | --- | --- | --- | --- |
| **Bro 07** | Amplification 1 | **228** | **230** | - | - | - | - | - | - | - | - | - | - | - | - | - | - | - | - | - | - | - | - | - | - | - | - | - |
|  | Amplification 2 | 228 | 230 | - | - | - | - | - | - | - | - | - | - | - | - | - | - | - | - | - | - | - | - | - | - | - | - | - |
|  | Amplification 3 | 230 | - | - | - | - | - | - | - | - | - | - | - | - | - | - | - | - | - | - | - | - | - | - | - | - | - | - |
|  | Amplification 4 | 228 | - | - | - | - | - | - | - | - | - | - | - | - | - | - | - | - | - | - | - | - | - | - | - | - | - | - |
| **Bro 08** | Amplification 1 | **208** | **214** | - | - | - | - | - | - | - | - | - | - | - | - | - | 188 | - | - | *213* | *-* | - | 194 | - | - | *201* | *213* | - |
|  | Amplification 2 | **202** | **208** | - | - | - | - | 194 | - | - | *201* | 208 | *215* | - | - | - |  | - | - | - | - | - | - | - | - | - | - | - |
|  | Amplification 3 | - | - | - | - | - | - | - | - | - | - | - | - | - | - | - | - | - | - | - | - | - | - | - | - | - | - | - |
|  | Amplification 4 | 202 | 208 | - | - | - | - | - | - | - | - | - | - | - | - | - | 202 | 208 | 210 | 202 | 208 | *213* | *213* | - | - | 202 | *222* | - |
| **Bro 13** | Amplification 1 | - | - | - | - | - | - | - | - | - | - | - | - | - | - | - | - | - | - | - | - | - | - | - | - | - | - | - |
|  | Amplification 2 | 220 | **226** | ***230*** | - | - | - | 222 | - | - | *219* | - | - | 220 | 226 | *230* | - | - | - | - | - | - | 222 | - | - | - | - | - |
|  | Amplification 3 | - | - | - | *206* | *-* | - | - | - | - | - | - | - | - | - | - | *205* | *216* | - | *216* | - | - | - | - | - | *208* | - | - |
|  | Amplification 4 | 226 | *230* | - | - | - | - | - | - | - | - | - | - | - | - | - | - | - | - | - | - | - | - | - | - | - | - | - |
| **Bro 15** | Amplification 1 | **210** | **214** | - | - | - | - | - | - | - | - | - | - | - | - | - | 221 | - | - | 221 | *223* | *232* | - | - | - | 213 | *232* | - |
|  | Amplification 2 | **210** | **214** | 221 | - | - | - | - | - | - | - | - | - | - | - | - | - | - | - | - | - | - | - | - | - | - | - | - |
|  | Amplification 3 | - | - | - | - | - | - | - | - | - | - | - | - | - | - | - | - | - | - | - | - | - | *196* | 222 | - | - | - | - |
|  | Amplification 4 | 210 | 214 | - | - | - | - | - | - | - | - | - | - | - | - | - | *207* | *-* | *-* | *192* | - | - | *192* | 202 | *226* | *192* | *231* | - |
| **Bropap 02214** | Amplification 1 | **241** | **247** | 249 | - | - | - | - | - | - | - | - | - | - | - | - | - | - | - | - | - | - | - | - | - | - | - | - |
|  | Amplification 2 | **241** | 245 | **247** | - | - | - | 239 | - | - | - | - | - | - | - | - | - | - | - | - | - | - | 239 | - | - | - | - | - |
|  | Amplification 3 | - | - | - | *221* | - | - | - | - | - | - | - | - | - | - | - | - | - | - | 193 | *202* | - | 227 | - | - | - | - | - |
|  | Amplification 4 | 241 | 247 | - | - | - | - | - | - | - | - | - | - | - | - | - | - | - | - | - | - | - | - | - | - | - | - | - |
| **Bropap 02801** | Amplification 1 | - | - | - | - | - | - | - | - | - | - | - | - | - | - | - | - | - | - | - | - | - | - | - | - | - | - | - |
|  | Amplification 2 | **149** | ***155*** | **168** | - | - | - | 147 | 149 | *167* | - | - | - | - | - | - | - | - | - | - | - | - | 147 | *156* | *-* | - | - | - |
|  | Amplification 3 | - | - | - | 147 | 159 | - | - | - | - | - | - | - | - | - | - | *137* | 140 | ***156*** | *148* | *156* | - | **140** | **147** | ***156*** | *144* | *146* | ***156*** |
|  | Amplification 4 | 149 | 168 | - | - | - | - | - | - | - | - | - | - | - | - | - | - | - | - | - | - | - | - | - | - | - | - | - |
| **Bropap 20558** | Amplification 1 | - | - | - | - | - | - | - | - | - | - | - | - | - | - | - | - | - | - | - | - | - | - | - | - | - | - | - |
|  | Amplification 2 | - | - | - | - | - | - | - | - | - | - | - | - | - | - | - | - | - | - | - | - | - | - | - | - | - | - | - |
|  | Amplification 3 | - | - | - | 219 | *222* | - | - | - | - | - | - | - | - | - | - | - | - | - | - | - | - | - | - | - | 219 | *233* | - |
|  | Amplification 4 | 217 | 221 | - | - | - | - | - | - | - | - | - | - | - | - | - | - | - | - | - | - | - | - | - | - | 219 | - | - |
| **Bropap 25444** | Amplification 1 | 173 | - | - | - | - | - | - | - | - | - | - | - | - | - | - | 185 | 189 | - | 185 | 189 | - | - | - | - | - | - | - |
|  | Amplification 2 | 185 | 188 | - | - | - | - | - | - | - | - | - | - | - | - | - | - | - | - | - | - | - | 179 |  | - | - | - | - |
|  | Amplification 3 | 185 | 188 | - | - | - | - | - | - | - | - | - | - | - | - | - | - | - | - | - | - | - | - | - | - | - | - | - |
|  | Amplification 4 | 179 | - | - | - | - | - | - | - | - | - | - | - | - | - | - | - | - | - | 179 | - | - | 173 | 185 | - | 179 | - | - |
| **Bropap 26985** | Amplification 1 | **177** | **182** | - | *173* | - | - | - | - | - | 177 | - | - | - | - | - | - | - | - | 177 | 184 | - | - | - | - | - | - | - |
|  | Amplification 2 | **177** | ***181*** | ***-*** | - | - | - | - | - | - | - | - | - | - | - | - | - | - | - | - | - | - | *181* | *203* | - | - | - | - |
|  | Amplification 3 | - | - | - | - | - | - | - | - | - | - | - | - | - | - | - | *195* | - | - | - | - | - | - | - | - | - | - | - |
|  | Amplification 4 | 177 | 182 | - | - | - | - | - | - | - | - | - | - | - | - | - | - | - | - | - | - | - | - | - | - | - | - | - |
| **Bropap 30248** | Amplification 1 | - | - | - | - | - | - | - | - | - | - | - | - | - | - | - | - | - | - | - | - | - | - | - | - | - | - | - |
|  | Amplification 2 | **93** | - | - | - | - | - | - | - | - | - | - | - | - | - | - | - | - | - | - | - | - | 99 | - | - | - | - | - |
|  | Amplification 3 | - | - | - | - | - | - | - | - | - | - | - | - | - | - | - | - | - | - | - | - | - | - | - | - | - | - | - |
|  | Amplification 4 | 93 | - | - | - | - | - | - | - | - | - | - | - | - | - | - | - | - | - | - | - | - | - | - | - | - | - | - |

Amplifications 1-3 were performed at the Faculty of Chemical and Pharmaceutical Sciences, University of Chile using GoTaq® G2 Flexi DNA Polymerase. Amplification 4 was performed at the Faculty of Medicine, University of Chile using GoTaq® G2 Hot Start Polymerase. In bold: alleles found in more than one replicate (extractions and PCR reactions) of the same sample using different enzymes; underlined: alleles previously detected exclusively in herbarium samples from Remote Oceania; in italics and undelined with dots: new alleles in textiles.
