# Supplementary material for: A tale of textiles: Genetic characterization of historical paper mulberry barkcloth from Oceania
Source: PLoS One. 2020 May 18;15(5):e0233113. doi: 10.1371/journal.pone.0233113 (PMC7233582; doi:10.1371/journal.pone.0233113)
Supplement: S1 Text — (DOCX) [file pone.0233113.s011.docx]

**S1 Text. Barkcloth descriptions.**

Sample from Rapa Nui barkcloth (accession number BQUCHTE001): This is a small piece of twisted fiber about one cm in length, used as cordage for decorative stitching on a large dyed tapa piece from the Bishop Museum collection (catalogue number D.2228). The provenance of the tapa is indicated as possibly from Rapa Nui. There is no information about the year of manufacture of the barkcloth or collection date.

Barkcloth samples from Hawaiian textiles (accession numbers BQUCHTE002- BQUCHTE003- BQUCHTE005-BQUCHTE006-BQUCHTE009-BQUCHTE010- BQUCHTE011): Sample BQUCHTE002 is a small piece of barkcloth (approximately 2 x 1 cm) from a large textile held at the Bishop Museum (catalogue number 2494). The fragment shows black lines on its surface, possibly of organic origin such as charcoal. Sample BQUCHTE003 was taken from a 5 x 10 cm unpainted rectangular sampler from the Bishop Museum collection that was cut off from a larger textile. It has no catalogue number and no information about the date of manufacture. Sample BQUCHTE005 measures ca. 1.5 x 1 cm and was taken from a large bedding barkcloth made of five barkcloth layers from the collection of the Honolulu Museum of Arts in Hawaii (catalogue number 662.1). Only the outer layer is decorated and is pink in color, produced by the inclusion of reddish fibers of an unknown material that were beaten into the bark fibers during the manufacturing process. This pink background is decorated with bands of small black square dots closely stamped together. The inner layers are natural undyed tapa. The sample is a small fragment from one of the inner layers. There is no information about the date of manufacture of this piece or when it was acquired by the Museum. Sample BQUCHTE006 was taken from a large decorated barkcloth that measures 62 x 350 cm, donated to the Honolulu Museum of Art in Hawaii in 1935 by Mrs. Charles M. Cooke (catalogue number 4121). This textile is either the outer layer of a bed tapa (*kapa moe*), or a tapa hanging. It is brown and decorated with a geometric design and a wide area of diamond-shaped motifs in darker brown. The textile is lined with brown muslin and backed with a water-marked sheet of plain tapa. The sample was taken from the outer layer and consists of several small fragments of 1.0 x 1.0 cm). We have no information on the manufacturing date of the piece. Sample BQUCHTE009 was obtained from a barkcloth from the Bishop Museum collection (catalogue number 2492). It is an unpainted fragment of 3.0 x 1.0 cm. Sample BQUCHTE010 is a small 1.0 x 3.0 cm fragment. The label of this textile indicated ‘Hawaii (?)’, with no further information. Barkcloth sample BQUCHTE011 measures 3.0 x 7.0 cm and was taken from an undecorated brown barkcloth textile. It is part of the Emory collection held at the Bishop Museum. There is no information about the year of manufacture or precise provenance of this textile.

Sample from New Guinea barkcloth (accession number BQUCHTE004): The sample size is approximately 5 x 2 cm and was taken from a Maisin textile from Oro Province, Papua New Guinea that is part of a private collection (T. Allen, Hawai’i). Manufacture of this piece is said to date to between 1980 and 1990. It is the youngest textile analyzed in this work.

Barkcloth samples from Fijian textiles (accession number BQUCTE007-BQUCTE008): Sample BQUCHTE007 measures approximately 10 cm x 3 cm and was obtained from a white undecorated barkcloth piece. Sample BQUCHTE008 was obtained from a decorated piece of barkcloth that measures 62 x 350 with dominantly black with rust and white designs. These designs are placed in three sections containing bands of various geometric patterns and a wide area of diamond-shaped motifs. The sample taken from this textile consists of several small fragments (4 x 0.5 cm) and shows black lines and red coloring on the surface. This textile belongs to the Honolulu Museum of Art in Hawaii (catalogue number 2506) and was donated to the Museum in 1928 by Mrs. Charles M. Cooke. We have no information of its manufacturing date.

Sample from a barkcloth textile from American Samoa (accession number BQUCHTE012): The size of this sample is approximately 4.5 x 2 cm and was obtained from a textile that is part of a private collection (T. Allen, Hawai’i). Manufacture of this piece is said to date to between 1880 and 1900. It is the second oldest piece analyzed in this work.

Samples from barkcloth textiles from the Gambier Islands (accession numbers BQUCH0013, BQUCH0014, BQUCH0015, BQUCH0016): These samples were obtained from a barkcloth bundle found in an archaeological context in a cave on Agakauitai Island, Gambier archipelago, French Polynesia, and has been described previously [20] It was radiocarbon dated to a *terminus ante quem* calibrated date of AD1834 ±5 AD. Samples referred to in Seelenfreund et al. [20] as BA1 and BA2 correspond to BQUCH0013 and BQUCH0014, respectively. Samples BB1 and BB2 correspond to accession numbers BQUCHTE016 and BQUCHTE015, respectively. These four samples will be referred to collectively as “Gambier Island” samples.
