# Supplementary material for: A tale of textiles: Genetic characterization of historical paper mulberry barkcloth from Oceania
Source: PLoS One. 2020 May 18;15(5):e0233113. doi: 10.1371/journal.pone.0233113 (PMC7233582; doi:10.1371/journal.pone.0233113)
Supplement: S2 Text — (DOCX) [file pone.0233113.s012.docx]

**S2. Text. Redesign and amplification of the sex marker region.**

The sex marker assay, previously used to identify contemporary and herbarium female and male specimens of *B. papyrifera* [23, 24] was redesigned and adapted to obtain smaller PCR amplicons (S6 Fig). The assay with the new primers Fw_Bp1: 5'- CCC GTA ACC GTG GAT CTA AAA AGC-3'; RC_MMRS18: 5'- TGA TGA CGT TGA CAT GGC-3' and MMRL: 5'- CTG GAC AAG ACC AAC TTT GAA TCC G-3' is shown in S6 Fig. The amplification protocol was as described by Payacán et al.[24] and the new design tested on contemporary DNA samples from visually typed female and male *B. papyrifera* individuals, and on DNA extracted from contemporary barkcloth (S6 Fig).

As controls, two samples from contemporary *B. papyrifera* leaves from Taiwan (accession numbers BQUCH0137 and BQUCH0140) from our genomic DNA bank were used. The amplified samples were analyzed by visual inspection on 1.5% agarose gels. Female samples displayed a single 300 bp band, while male samples exhibited two bands of 300 and 160 base pairs.

**Results**

A sex marker assay designed for *B. papyrifera* was used to analyze DNA obtained from all textiles samples. As shown in S7 Fig, only the sample from New Guinea (BQUCHTE0004) could be identified as originating from paper mulberry bark from female specimens. None of the other samples could be analyzed with this marker.

We adapted the sex marker assay previously reported [23] to obtain smaller amplicons, of approximately 300 bp in female specimens (instead of 420 bp) and two 300 bp and 165 bp fragments in male specimens (instead of 420 and 273 bp) (S6 Fig). In this case, just one sample (the most contemporary one) could be amplified using this marker (S7 Fig).
