# Supplementary material for: A tale of textiles: Genetic characterization of historical paper mulberry barkcloth from Oceania
Source: PLoS One. 2020 May 18;15(5):e0233113. doi: 10.1371/journal.pone.0233113 (PMC7233582; doi:10.1371/journal.pone.0233113)
Supplement: S3 Text — (DOCX) [file pone.0233113.s013.docx]

**S3 Text. Permits**

Export of samples from Tahiti to Chile was possible through permit 548/2014 issued by the Service de la Culture et du Patrimoine of French Polynesia to AS. Samples from the B. P.Bishop Museum, Honolulu, Hawai, USA and the Honolulu Museum of Art, Honolulu, Hawai, USA, were provided by the respective collection curators to AS.
